# Higher-Throughput Proteome Profiling Enabled by Parallelized Pre-Accumulation and Optimized Ion Processing in the Orbitrap Astral Zoom Mass Spectrometer

**DOI:** 10.1016/j.mcpro.2025.101504

**Published:** 2026-01-09

**Authors:** Ulises H. Guzman, Martin Rykær, Ivo A. Hendriks, Hamish Stewart, Eduard Denisov, Bernd Hagedorn, Johannes Petzoldt, Arne Kreutzmann, Yannick Mueller, Tabiwang N. Arrey, Immo Colonius, Ole Østergaard, Claire Koenig, Julia Kraegenbring, Kyle L. Fort, Erik Couzijn, Jan-Peter Hauschild, Daniel Hermanson, Vlad Zabrouskov, Christian Hock, Eugen Damoc, Jesper.V. Olsen

**Affiliations:** 1 Department of Cellular and Molecular Medicine, Novo Nordisk Foundation Center for Protein Research, Faculty of Health and Medical Sciences, University of Copenhagen, Copenhagen, Denmark; 2 Thermo Fisher Scientific, Bremen, Germany

**Keywords:** Orbitrap Astral Zoom, high-throughput proteomics, evosep ENO, narrow-window DIA, enhanced dynamic range scans

## Abstract

High-throughput proteomics is critical for understanding biological processes, enabling large-scale studies such as biomarker discovery and systems biology. However, current mass spectrometry technologies face limitations in speed, sensitivity, and scalability for analyzing large sample cohorts. The Thermo Scientific Orbitrap Astral Zoom mass spectrometer (MS) was developed to address these limitations by improving acquisition speed, ion utilization, and spectral processing, which are all essential for advancing proteome depth in high-throughput proteomics. The Orbitrap Astral Zoom MS achieves ultra-fast MS/MS scan rates of up to 270 Hz with enhanced ion utilization through pre-accumulation, enabling the identification of ∼100,000 unique peptides and >8400 proteins in a single 300 samples-per-day analysis of human cell lysate. The optimized system reduces analysis time by 40%, achieves near-complete proteome coverage (>12,000 proteins) in 2.7 h, and enables ultra-high-throughput workflows, identifying >7000 proteins in a 500 samples-per-day method with exceptional reproducibility (pairwise Pearson correlations >0.99). These advancements establish the Orbitrap Astral Zoom MS among the fastest and most sensitive instruments under the tested conditions, significantly enhancing speed, sensitivity, and scalability, paving the way for routine large-scale proteomics with applications in clinical research and systems biology.

## Highlights

• Orbitrap Astral Zoom MS enables up to 270 Hz MS/MS with higher sensitivity.
• 500 SPD workflows identify >7,000 proteins with high reproducibility.
• 500 SPD runs maintain stable performance across >500 consecutive injections.
• Single 300 SPD analysis identifies ∼100,000 unique peptides.

## Graphical Abstract

**Figure:**
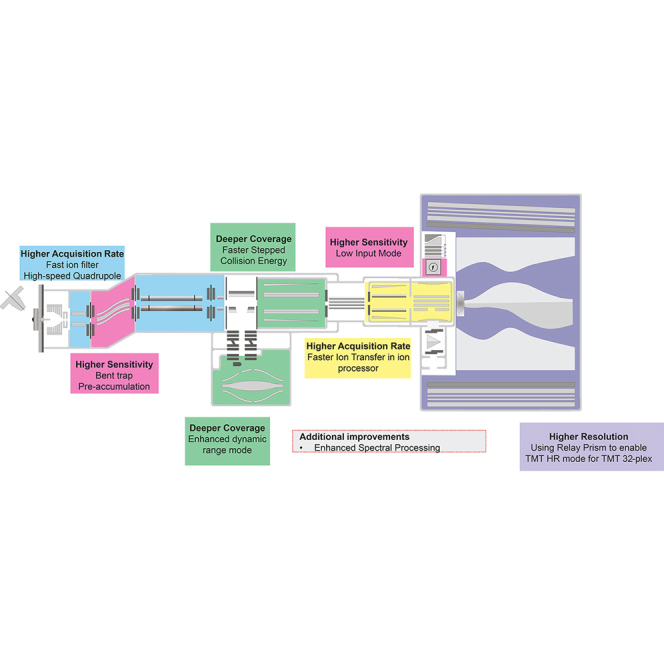

## In Brief

Guzman *et al*. characterize the performance of the Orbitrap Astral Zoom MS for high-throughput proteomics and benchmark its performance with that of the previous-generation Orbitrap Astral MS. Their data demonstrates advantages for very fast LC gradients of 300 and 500 samples-per-day with very high human proteome coverage and good quantitative reproducibility.

Biological systems are complex networks essential for organismal function, ranging from individual cells to complex ecosystems. They are inherently dynamic and their rapid response to perturbations are influenced by a variety of factors, such as genetic background and environmental conditions (1, 2). To understand their behavior, it is essential to systematically assess the proteome states across diverse populations, disease contexts, and model systems under varying conditions (3, 4).

Proteomics based on peptide sequencing by online liquid chromatography tandem mass spectrometry (LC-MS/MS) is generally the method of choice for such applications. Recent technological advancements in mass spectrometry-based proteomics technologies have facilitated remarkable proteome depth, sensitivity, and throughput (5, 6, 7, 8). However, systems biology studies, biomarker discovery from large clinical research cohorts, or drug library screenings by mass spectrometry-based proteomics are dependent on deep proteome coverage with high reproducibility across hundreds or thousands of samples. Therefore, further scaling of such proteomics experiments is crucial, not only to decrease MS measurement time but also to generate high-quality, comprehensive datasets suitable for advanced statistical analyses and modeling, including machine learning (9, 10).

Traditionally, comprehensive mass spectrometry-based proteomics projects require days, weeks, or even months of LC-MS/MS instrument time. Addressing these demands requires the shortening of chromatographic gradients, while preserving both sensitivity and proteome coverage. As a result, mass spectrometers must operate at higher acquisition rates to process the same number of analytes within reduced time frames (11), while ensuring sufficient selectivity to resolve co-eluting peptides and preserve quantitative performance. State-of-the-art proteomics-grade MS systems operated in data-independent acquisition (DIA) mode are capable of acquiring more than one hundred high-resolution accurate mass DIA-MS/MS scans per second, which enabled the development of fast acquisition methods (12). For instance, the Thermo Scientific Orbitrap Astral MS can achieve acquisition rates of ∼200 Hz (13, 14, 15) and narrow (2 Th) isolation windows that have proven effective for DIA applications, despite reduced utilization of the ion beam (8).

Although these capabilities are significant, further improvements in speed and sensitivity can be achieved by optimizing the ion processing stage and further parallelizing ion handling during the instrument's overheads. The operation of the Orbitrap Astral MS is highly parallelized, with multiple ion packets processed simultaneously to decouple ion accumulation time from downstream processing stages. Nevertheless, it still suffers from almost 2 ms scan-to-scan overhead, which substantially impacts duty cycle at maximum 200 Hz operational scanning speed equivalent of ∼5 ms acquisition time per MS/MS spectrum (13). The scan-to-scan overhead therefore makes up ∼40% of the cycle time at the fastest scanning DIA methods, limiting ion accumulation time to 3 ms at 200 Hz acquisition rates.

Here, we present an optimized ion processing stage and enhanced spectral processing algorithm (16) on an Orbitrap Astral Zoom MS. The improved ion processing including pre-accumulation (pre-AC) of ions in the bent trap facilitates higher acquisition rates and shorter duty cycle while maintaining or increasing ion count per scan. In addition, an enhanced spectral processing algorithm implemented across the whole mass range improves the deconvolution of overlapping spectral features in MS/MS scans to better resolve feature-rich spectra, increasing the number of detectable fragment ions in MS/MS spectra. Collectively, these hardware and software improvements enable ultra-fast DIA-MS/MS scans rates of up to 270 Hz, preserving the high selectivity of narrow-window DIA without sacrificing ion accumulation time and spectral quality.

Compared to the Orbitrap Astral MS (8), the Orbitrap Astral Zoom MS identified more peptides and proteins in half the LC gradient time with ∼100,000 unique peptides and ∼8400 protein-coding genes (hereafter referred to as protein groups or PGs) with a 300 samples-per-day (SPD) method and >7000 unique proteins from a 500 SPD LC-MS/MS method. Similar improvements in analytical depth were observed with extended gradients, such as 24 SPD, enabling the identification of >10,200 protein groups and >200,000 unique peptides. Furthermore, these optimizations accelerated the acquisition of near-complete proteomes (>12,000 PGs), reducing the analysis time for 34 high-pH fractions by 40%. This advancement allowed for the acquisition of a full proteome in ∼3 h. This approach also provides sufficient depth to confidently identify and localize more than 2000 phosphosites without enrichment, as well as ∼ 1600 splicing variants.

## Experimental Procedures

### Sample Preparation

#### Cell Lines

All human cell lines were cultured in Dulbecco’s modified Eagle medium (Gibco, Invitrogen), supplemented with 10% fetal bovine serum, 100 U/ml penicillin (Invitrogen), and 100 μg/ml streptomycin (Invitrogen). The cell cultures were maintained at 37 °C, in a humidified incubator with 5% CO_2_. Cells were collected at approximately 70% confluence by washing twice with PBS (Gibco, Life technologies). Subsequently, boiling lysis buffer (5% SDS, 5 mM Tris(2-carboxyethyl)phosphine, 10 mM chloroacetamide, 100 mM Tris, pH 8.5) was added directly to the plates. The cell lysate was collected by scraping the plates and boiled for an additional 10 min. Following this, micro-tip probe sonication (Vibra-Cell VCX130, Sonics) was performed for 2 min with pulses of 1 s on and 1 s off at 80% amplitude. Protein concentration was determined by bicinchoninic acid protein assay.

#### Preparation of Samples for LC–MS/MS Analysis

The human cell lines were digested overnight at 37 °C with looping mixing, employing the protein aggregation capture protocol (17) with MagReSyn amine microparticles (ReSyn Biosciences) on a fully automated KingFisher platform. Briefly, 1 mg of protein lysate was resuspended in acetonitrile (ACN) to a final concentration of 70%. MagReSyn amine microparticles were added in a proportion 1:2 (protein:beads). The proteolytic digestion was performed by the addition of lysyl endopeptidase (LysC, Wako) and trypsin enzymes at 1:500 and 1:250 protease-to-protein ratio, respectively, to 300 μl of digestion buffer (50 mM ammonium bicarbonate). Protein aggregation was carried out in two cycles, each consisting of 1 min mixing at 1000 rpm followed by a 10 min pause. The beads were subsequently washed three times with 1 ml 95% ACN and two times 1 ml 70% ACN. The resulting peptide mixture was acidified with TFA to a final concentration of 1% to quench the protease activity. Finally, the peptides were concentrated by a Sep-Pak C18 96-well Plate (Waters), with peptides eluted sequentially using 150 μl of 40% ACN followed by 150 μl of 60% ACN and collected in a 96-well plate. The combined eluate was dried down via SpeedVac vacuum concentrator (Eppendorf). Finally, the peptide mixture was resuspended in 100 μL 0.1% TFA, and the final peptide concentration was determined by measuring absorbance at 280 nm on a Thermo Scientific NanoDrop 2000C spectrophotometer. The protein digests for the mixed species quantitative analysis were purchased from Pierce for HeLa (88328), Promega for yeast (V7461), and Waters for *Escherichia coli* (SKU: 186003196). They were manually mixed in three different ratios, E05-H50-Y45, E45-H50-Y05, and E25-H50-Y25, respectively. Samples were kept at −20 °C until analysis

#### Off-Line HpH Reversed-Phase HPLC Fractionation

HEK293 peptides (200 μg) were separated by high-pH (HpH) reversed-phase chromatography using a reversed-phase Acquity CSH C_18_ 1.7 μm × 1 mm × 150 mm column (Waters) on a Thermo Scientific UltiMate 3000 HPLC system with the Chromeleon software. The instrument was operated at a flow rate of 30 μl min^−1^ with buffer A (5 mM ammonium bicarbonate) and buffer B (100% ACN). Peptides were separated by a multistep gradient as follows: 0 to 10 min 6.5% B to 15% B, 10 to 59.5 min 15% B to 30% B, 59.5 to 67 min 30% B to 65% B, 67 to 70 min 65% B to 80% B, 70 to 77 min 80% B, 78 to 87 min 6.5% B. A total of 46, 24, and 12 fractions were collected at 60 s intervals. Samples were acidified using 30 μl of 10% formic acid. The samples were dried down using a SpeedVac vacuum concentrator. Sample concatenation was performed manually. Two hundred nanograms of each fraction were injected for LC–MS/MS analysis.

#### LC–MS/MS Analysis

LC–MS/MS analysis was performed on an Orbitrap Astral Zoom MS prototype coupled to a Thermo Scientific Vanquish Neo UHPLC or an Evosep ENO system and interfaced online using an EASY-Spray/Nano-Flex ion source. Depending on the gradient utilized, different setups were employed, including either trap-and-elute or direct injection into commercial or home-packed analytical columns. The choice of column type was made according to the gradient employed (Supplemental Table S2). A blank run of equal gradient length was run after three or four runs. The 300-SPD and 500-gradient methods were run on an Orbitrap Astral Zoom MS prototype, coupled to a Thermo Scientific Vanquish Neo UHPLC and an Evosep ENO system, respectively. For the 300-SPD method, the active gradient was ∼216 s; hardware and software overheads were 84 s and 21 s respectively for a total run-to-run time, including both hardware and software overhead of ∼321 s (∼270 SPD). For the 500-SPD method, the active gradient was 138 s, hardware and software overhead were 35 s and 30 s respectively for a total run-to-run time, of ∼203 s (∼426 SPD).

For the data-dependent acquisition (DDA) experiments, the Orbitrap Astral Zoom MS prototype was operated with a fixed cycle time of 0.6 s and with a full scan range of 400 to 900 *m*/*z* at a resolution of 240,000. The automatic gain control (AGC) target value for the Orbitrap analyzer was set to 5e6. Precursor ion selection width was kept at 2 Th and peptide fragmentation was achieved by higher-energy collisional dissociation, with normalized collision energy at 30%. Fragment ion scans were recorded at a resolution of 80,000 @ m/z 524, with a maximum fill time (maxIT) of 3 ms. Dynamic exclusion was enabled and set to 10 s. The AGC target value for the Astral analyzer was set to 2e4. In wide window data-dependent acquisition experiments, the same settings were applied, except the precursor ion selection width was maintained at 4 Th, and fragment ion scans were recorded with a maxIT of 6 ms. When the enhanced dynamic range mode was used, five symmetric MS1 windows of 100 Th each were configured, covering the range of 400 to 900 *m/z*, with an AGC target value of 5e6 and a maxIT of 50 ms on the Orbitrap analyzer. The precursor ion selection width was set to either 2 Th or 4 Th, and MS/MS spectra were recorded with maxIT values of 3 ms and 6 ms, respectively.

For the DIA experiments, an Orbitrap Astral Zoom MS prototype was operated at a full-MS resolution of 240,000 with a full scan range of 380 to 980 *m/z* otherwise stated. The full-MS AGC target value was set to 5e6. Fragment ion scans were recorded at a resolution of 80,000 @ m/z 524 and maxIT of 1.5 ms. We used 300 windows of 2 Th scanning from 380 to 980 *m*/*z*, unless stated otherwise in Supplemental Table S2. The isolated ions were fragmented using higher-energy collisional dissociation with 25% normalized collision energy. For the HEK293 measured at 500 SPD in the Orbitrap Astral mass spectrometer, peptides were eluted online from the EvoTip using an Evosep ENO system (Evosep Biosystems) using a commercial -cm analytical column (Evosep Endurance EV-1107 4 cm × 150 μm, 1.9 μm).

Further details are described in Supplemental Table S2.

#### Raw MS Data Analysis

Raw files from DIA and DDA comparison experiments were analyzed in DIA-NN v1.9.2 (18) software, allowing for C carbamidomethylation and N-terminal M excision and one missed cleavage (default configuration). The spectral library was generated from a human reference database (UniProt 2024 release, 20,417 canonical sequences). Raw files from single-shot dilution series of HEK peptides, optimization, and cell lines were analyzed in Spectronaut v19 (Biognosys) with a library-free approach (directDIA+) using the human reference database (UniProt 2024 release, 20,417 canonical sequences) complemented with common contaminants (246 sequences). Cysteine carbamylation was set as a fixed modification, whereas methionine oxidation and protein N-terminal acetylation were set as variable modifications. Precursor filtering was set as *Q* value, and cross-run normalization was unchecked. Each experiment was analyzed separately, and those that contained different technical conditions (different input amounts or acquisition methods) were searched with method evaluation enabled, with the different conditions (each one with *n* = 4 experimental replicates) indicated in the condition setup tab.

Raw files from label-free quantification (LFQ) analysis of the mixed species samples were analyzed in Spectronaut v19 (Biognosys) with a library-free approach (directDIA+) using a benchmark reference database for the three species (31,657 sequences in total). Cysteine carbamylation was set as a fixed modification, whereas methionine oxidation and protein N-terminal acetylation were set as variable modifications. Precursor filtering was set as *Q* value, and cross-run normalization was enabled. Experiments with identical sample loading amounts were analyzed separately, with each condition including N experimental replicates. All other search parameters were set to default.

Raw files from different fractionation schemes were analyzed in Spectronaut v19 (Biognosys) with a library-free approach (directDIA+) using the human reference database (UniProt 2024 release, 20,417 canonical sequences) complemented with common contaminants (246 sequences). Methionine oxidation and protein N-terminal acetylation were set as variables, whereas cysteine carbamylation was set as a fixed modification. Precursor filtering was set as *Q* value. Each fractionation scheme was searched independently, except for searches performed in triplicate. Quantification was performed using the MaxLFQ algorithm embedded in iq R package (19). Briefly, extended Spectronaut output results were filtered as follows: PG.Qvalue <0.01 and EG.Qvalue <0.01. Then, the MaxLFQ algorithm was applied using PG.Genes and PG.ProteinNames for protein annotation. Finally, to determine the percentages of residues in each identified protein sequence (sequence coverage), the program protein coverage summarizer was used. The human reference database used for quantification and a file containing all detected peptide sequences with a protein name associated (PG.Qvalue <0.01 and EG.Qvalue <0.01) were utilized for protein assembly and sequence coverage calculation. The phosphopeptide search was performed using a phosphopeptide library (12 fractions, 119,793 precursors, with a site localization probability threshold of ≥0.75). This approach was chosen to improve data analysis speed in Spectronaut (SN v18), though the analysis can also be performed using a direct DIA search. Standard method settings were applied, including a 1% false discovery rate (FDR) for protein groups, peptides, and modified peptides, with phosphorylation of serine, threonine, and tyrosine (S, T, Y) specified as variable modifications and the site localization probability threshold was set to ≥0.75; Data were analyzed with Spectronaut v18 to enable direct comparison with reference datasets reported in Guzman, 2024. Spectronaut output was reformatted using the Perseus plugin (20) peptide collapse to create a MaxQuant-like site-table. Mass spectrometry statistical plots were based on MS1 feature detection output retrieved by MaxQuant (v1.6.7.0) or MaxQuant (v1.6.14.0). Representative raw files for each method were loaded into MaxQuant and analyzed without the indication of a FASTA file, to extract only the relevant MS features. Enrichment analysis was performed with the package clusterProfiler (21) using the org.Hs.eg.db. All data analysis was performed using R v4.2.2 and R studio v2022.12.0 Build 353. The proteogenomics workflow was performed following Chamber *et al*., 2017 (22), using the HEK transcriptome available from public databases (Malm *et al*., 2020 (23)) and the Galaxy guidelines. The HEK transcriptome was used to generate a variant FASTA file containing 445,174 sequences, including splice variants, single amino acid variants (SAVs), and small indels. Searches were performed requiring PG.Qvalue <0.01 and EG.Qvalue <0.01 and at least two precursors identified to ensure confidence in variant identifications. The complete variant FASTA and the full proteogenomic analysis pipeline are provided in the Data availability section. Library-free SN v19 analysis was performed using standard settings, including a peptide length range of 7 to 52 amino acids, allowance for up to two missed cleavages, a precursor charge range of 1 to 4, a precursor m/z range of 200 to 3000, and a minimum of three fragments per peptide. A 1% FDR threshold was applied at the precursor, peptide, and protein group levels. The human proteome FASTA file used for the analysis was downloaded from UniProt on 13 November 2024.

DIA-NN analysis was performed using v1.9.2 (18), either using an in silico predicted library (library fee) or with an empirical library. All DIA-NN output files were filtered for protein or peptide Q-value to approach 1% FDR. For evaluation of high-throughput gradients (300 and 500 SPD), the GG_Matrix and Summary output was used to generate the Pearson correlations and Unique gene counts.

**Fig. 1:**
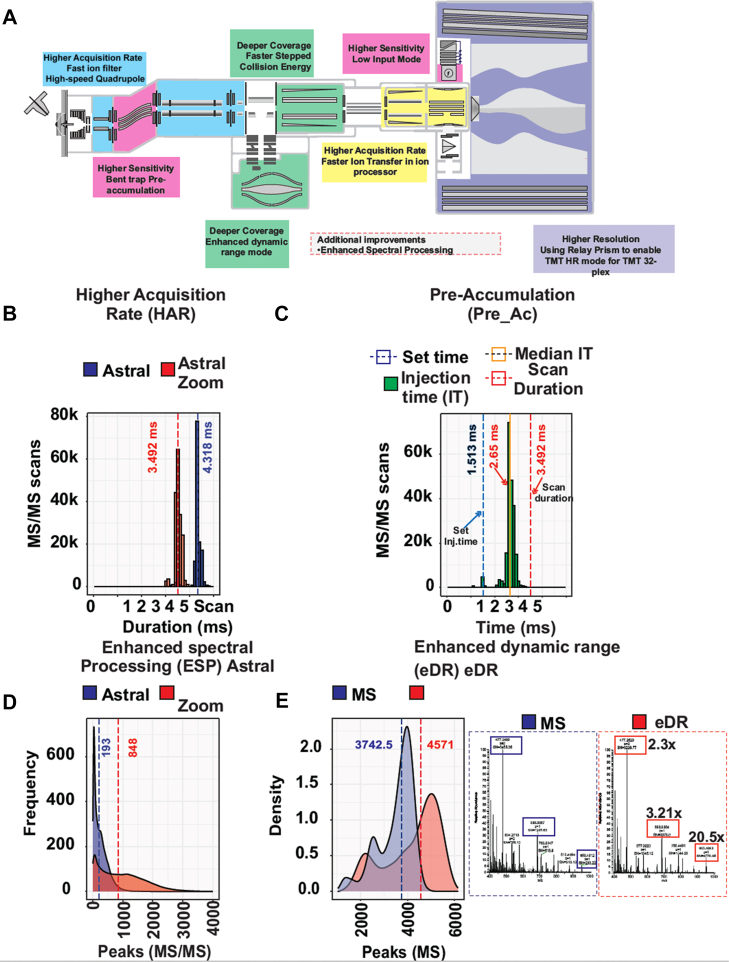
**Schematic of the Orbitrap Astral Zoom mass spectrometer and enhanced ion processing features**. *A*, hardware overview of the Orbitrap Astral Zoom (OAZ) instrument, highlighting new features over the Orbitrap Astral MS (OA). Median values are indicated by *dashed* lines. *B*, comparison of MS/MS median scan durations for OA (*blue*, *dashed*) and OAZ (*red*, *dashed*), where OAZ was acquired with high acquisition rate (HAR) enabled. The distribution of OAZ MS/MS scan durations is shown in *red*, and the corresponding maximal injection-time distribution is shown in *blue*. *C*, effective injection time (IT) on the OAZ with the pre-accumulation feature (Pre-Ac) enabled (*green*). The *orange dashed* line indicates the median IT, whereas the *blue* and the *red dashed* lines indicate the set IT and the median scan duration. *D*, density plot showing the number of peaks detected in OA and OAZ MS/MS scans with the enhanced spectral processing (ESP) algorithm enabled; striped lines show the median peak counts per scan. *E*, (*Left*): density plot showing the total number of peaks detected in MS1 scans in OA and OAZ with the enhanced dynamic range (eDR) feature enabled. (*Right*): Representative MS1 scans illustrate the improvement in signal-to-noise (S/N) when eDR is enabled in OAZ compared to OA.

**Fig. 2:**
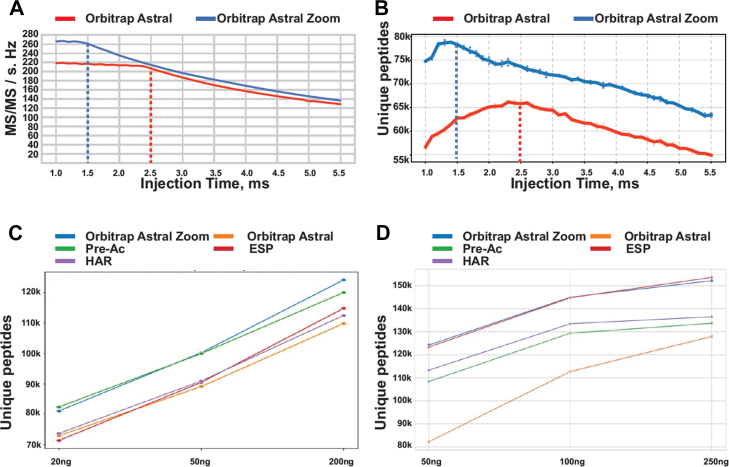
**Individual benchmarking of performance-enhancing features in the Orbitrap Astral Zoom mass spectrometer**. *A*, MS/MS scan rate as a function of ion injection time for the Orbitrap Astral Zoom MS and Orbitrap Astral MS. Optimal injection times for each system are indicated by *dashed* lines (300 SPD). *B*, number of unique peptides identified as a function of ion injection time for the Orbitrap Astral Zoom MS and Orbitrap Astral MS, with optimal values indicated by *dashed* lines (300 SPD; data processed with DIA-NN v1.9.2). *C*, contribution of individual hardware and software features to peptide identification yield using fast LC gradients (90 SPD; data processed with DIA-NN v1.9.2), with injection time restricted to 1.5 ms and 2 Th DIA isolation windows. Shown are the Orbitrap Astral Zoom MS (*blue*), Orbitrap Astral MS (*orange*), pre-accumulation (Pre-Ac; *green*), enhanced spectral processing (ESP; *red*), and high acquisition rate mode (HAR; *violet*). *D*, same analysis as in (*C*), performed using longer LC gradients (50 SPD; data processed with DIA-NN v1.9.2), with injection time restricted to 6 ms and 4 Th DIA isolation windows.

**Fig. 3:**
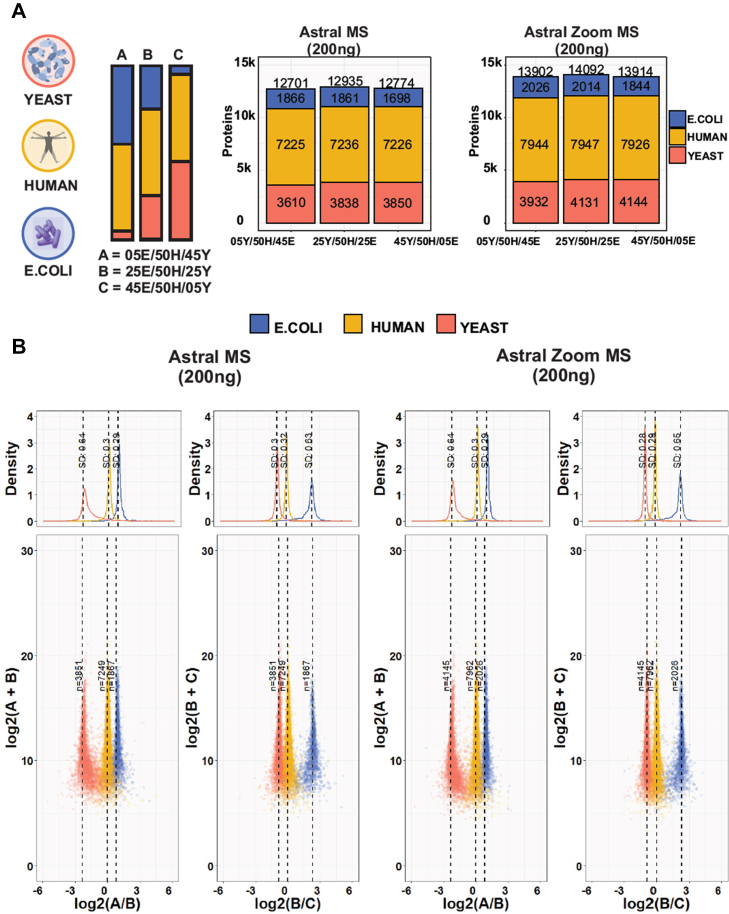
**Quantitative assessment of LFQ accuracy and precision on the Orbitrap Astral Zoom MS under high-speed scanning regimes**. *A*, graphical representation of the experimental design. Tryptic peptides from three species were combined in three distinct ratios (05E/50H/45Y, 25E/50H/25Y, and 45E/50H/05Y) (*left panel*). Number of proteins identified from the three species in each sample, for the Orbitrap Astral MS (*centerpanel*) and Orbitrap Astral Zoom MS respectively (*right panel*). Samples were processed using the Orbitrap Astral MS and Orbitrap Astral Zoom MS in technical triplicates, employing a 1.5-ms maxIT and 2-Th window size method. The loading amounts were 200 ng. *B*, log-transformed ratios of quantified proteins. Scatter plots for all runs over the log-transformed protein intensities are displayed at the *bottom*, while density plots are on the *top*. Colored dashed lines represent expected log2-fold-change values for proteins from humans (*yellow*), yeast (*orange*), and *E*. *coli* (*blue*). SDs are displayed on the density plots.

**Fig. 4:**
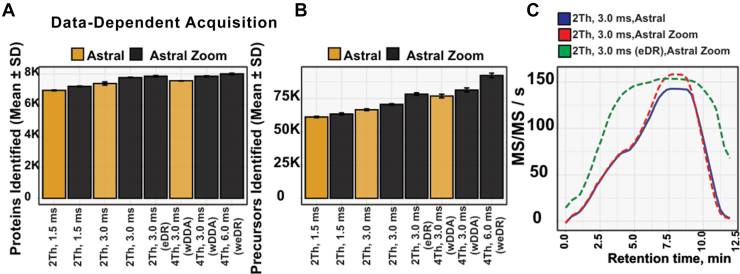
**Benchmarking enhanced MS1 dynamic range in DDA mode.***A*, data-dependent acquisition (DDA) performance at the protein group level comparing the Orbitrap Astral Zoom MS and the Orbitrap Astral MS at 100 samples per day (SPD) using 10 ng HEK293 tryptic peptides across four MS methods, with and without using enhanced dynamic range (eDR) mode. *B*, corresponding analysis at the peptide level. *C*, MS/MS scan rate (scans per second) over the LC gradient. (Data were processed using DIA-NN v1.9.2).

**Fig. 5:**
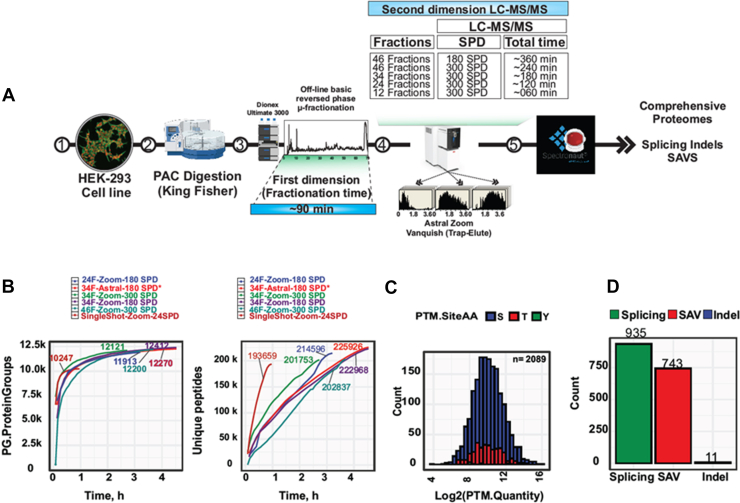
**High-coverage human proteome profiling via optimized multi-shot fractionation and fast LC-MS workflows**. *A*, schematic of the experimental workflow illustrating the fractionation strategies optimized for comprehensive proteome coverage. *B*, cumulative number of protein groups and peptides identified (*right* and *left panels*, respectively) as a function of total MS acquisition time including LC overhead. Shown are results from 46 and 34 high-pH reversed-phase (HpH) fractions acquired at 300 SPD, 34 and 24 HpH fractions acquired at 180 SPD, and single-shot analysis using a 24 SPD gradient on the Orbitrap Astral Zoom MS. For comparison, 34 HpH fractions acquired at 180 SPD using the Orbitrap Astral MS are also included (Guzman *et al*., 2024). *C*, sequence logo and abundance distribution of phosphorylation sites identified without enrichment. *D*, bar chart showing the number of detected splicing events, single amino acid variants (SAVs), and insertions/deletions (indels) (Data processed with SN v18).

**Fig. 6:**
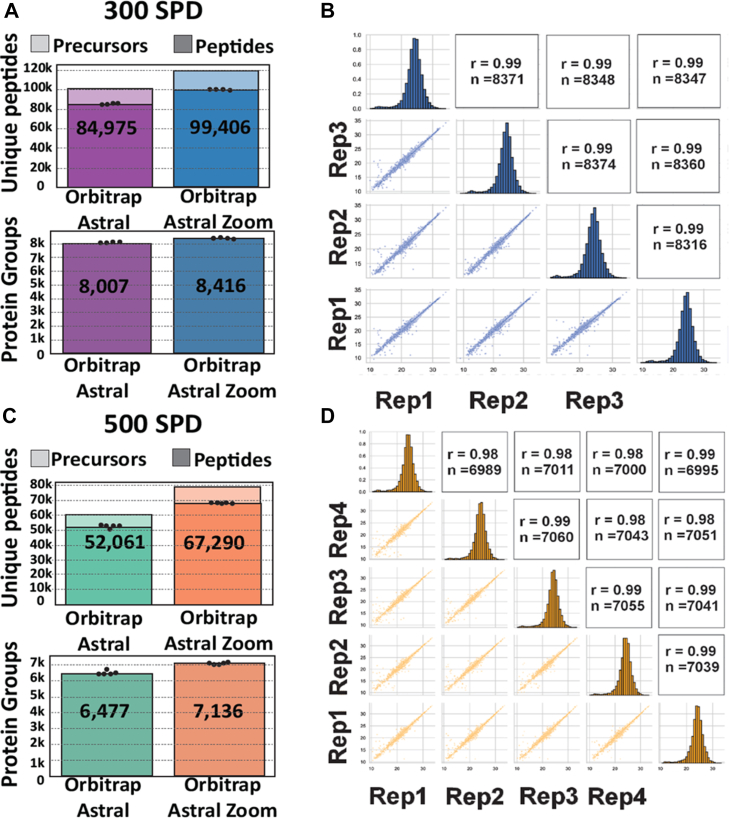
**Ultra-fast and deep proteome profiling with Orbitrap Astral Zoom MS and short-gradient LC**. *A*, number of unique peptides, precursors (*top*), and protein groups (*bottom*) identified from 200 ng HEK293 digest analyzed with a 300 SPD trap-and-elute Vanquish Neo LC gradient using the Orbitrap Astral Zoom MS. *B*, quantitative reproducibility of protein intensities across three technical replicates at 300 SPD. Shown are pairwise Pearson correlation coefficients (*upper triangle*), log_2_ intensity histograms (*diagonal*), and scatterplots of protein intensities between replicate pairs (*lower triangle*). *C*, same as in (*A*) but using a 500 SPD method on the Evosep Eno LC system coupled to the Orbitrap Astral Zoom MS. *D*, same as in (*B*), showing reproducibility across four technical replicates at 500 SPD. (Data processed with DIA-NN v2.0).

For analysis using an in silico library generated by DIA-NN (i.e. library-free), the standard settings were kept; peptide length 7 to 30, precursor charge range 1 to 4, precursor m/z range 300 to 1800, fragment m/z range 200 to 1800, 1% precursor FDR, with “No shared spectra” and “heuristic protein inference” enabled. The human proteome FASTA file was downloaded from Uniprot on the 13th of November 2024.

For analysis using an empirical library, the library was generated with DIA-NN by searching the 24, 34, 46 fraction experiment using an in silico predicted library and match between runs enabled. This empirical library was used for DIA-NN analysis with the same settings as described in the library-free method, except match between runs was turned off.

The three species mixes analyzed with 300 SPD and 500 SPD methods were analyzed using DIA-NN v2.0.

Scan intensity output, injection time (IT)s, scan counts, peak per scans were extracted using in-house scripts reading directly from the .raw file using Thermo Raw File Reader through Python.

## Results

### Optimized Ion Processing Stage Enables Increased Ion Beam Utilization and Faster MS/MS Acquisition

The Orbitrap Astral mass spectrometer combines a quadrupole with both a Thermo Scientific Orbitrap analyzer and a Thermo Scientific asymmetric track lossless (Astral) analyzer providing >200 Hz MS/MS scanning speed, high resolving power, high sensitivity, and low-ppm mass accuracy. This configuration enables the sensitivity and acquisition speed to routinely perform DIA analyses with quadrupole isolation window width similar to DDA, blurring the contrast between DIA and DDA (8).

Here, we introduce the Orbitrap Astral Zoom MS, which incorporates hardware and software enhancements that increase MS/MS scan speed, ion beam utilization and sensitivity, and improve the MS/MS peak deconvolution across the full m/z range (Fig. 1*A*; Supplemental Table S1). Briefly, a faster ion filter quadrupoles (blue) have been introduced to reduce scan overhead, enhancing the ion beam utilization with increased sensitivity. A parallel ion pre-Ac stage has been added in the bent trap (pink) upstream of the mass selection quadrupole. Stronger ion shuttling axial fields in the ion routing multipole (IRM) and more stable control hardware were implemented to reduce timing delay overheads. Moreover, faster ion transfer in the ion processor (yellow) has been implemented to increase acquisition rates, and the introduction of a relay prism in the Astral analyzer (purple) facilitates higher-resolution scans.

Collectively, the ion shuttling modifications resulted in optimized ion processing throughout the Orbitrap Astral Zoom MS compared to the Orbitrap Astral MS (Supplemental Fig. S1*A*); from the inject flatapole, through the bent trap, mass filter quadrupole, IRM, ion processor, and Astral analyzer. The enhanced ion processing reduced the median Astral DIA-MS/MS scan-to-scan time from 4.32 ms in the Orbitrap Astral to 3.49 ms in the Orbitrap Astral Zoom, enabling 270 Hz acquisition rates (Fig. 1*B*). Moreover, the pre-Ac stage occurring in the bent trap (a curved quadrupolar printed circuit board–mounted ion guide with a superimposed direct current gradient generated by a printed circuit board–printed electrode series (24)) operates concurrently, while downstream devices process the previous ion packet in an on-demand AGC fashion (Fig.1*C*, Supplemental Fig. S1*B*).

The pre-Ac approach adds approximately 1 ms of ion accumulation without increasing the overall cycle time. This enhances duty cycle and sensitivity at constant scan speed, while sustaining sensitivity at higher MS/MS acquisition rates. In addition, an enhanced spectral processing (ESP) algorithm was implemented across the whole mass range to deconvolute overlapping spectral features in MS/MS scans to better resolve feature-rich spectra, rather than rely entirely on the analyzer’s high resolving power to baseline separate different m/z peaks (Supplemental Fig. S1*C*). Briefly, this method segments the ion signal trace based on arrival times, applying adaptive filters tuned to expected ion arrival distributions to each segment, identifying ion peaks in the filtered data, thereby reducing misinterpretation of multimodal peaks as overlapping ions (16). Enabling this feature on the Orbitrap Astral Zoom MS resulted in a median 4-fold increase in the number of MS/MS spectral peaks compared to the Orbitrap Astral MS when using 300 SPD gradients (Fig. 1*D*).

Finally, to extend the dynamic range of Orbitrap MS1 scans, an enhanced dynamic range (eDR) mode acquisition strategy was implemented. Typically, intense ion populations that fill the IRM and transverse the C-trap can hinder detection of low-abundance analytes. The eDR feature overcomes this limitation by multiplexing injections with variable ion-accumulation times across different MS1 m/z ranges (Supplemental Fig. S1*D*). This approach selectively attenuates high-intensity m/z regions while amplifying low-intensity species. By segmenting the MS1 survey scan into predefined m/z ranges with tailored accumulation targets, eDR increases the number of detected peaks and enhances the signal-to-noise ratios of trace analytes. Using the eDR mode on the Orbitrap Astral Zoom MS resulted in up to a 20-fold improvement in signal-to-noise compared to the standard MS1 full scan and increased the median number of observed peaks in MS1 full scan by ∼22%, from 3742 to 4571 (Fig. 1*E*).

### Benchmarking the Impact of Hardware and Software Enhancements with the Orbitrap Astral Zoom MS

To determine the optimal ion IT for maximizing scan speed and ion beam utilization, we performed a titration experiment in which the IT in the IRM was incrementally increased from 1.0 to 5.5 ms in 0.1 ms steps. The instrument was operated in nDIA mode with 2 Th quadrupole isolation over an m/z range of 380 to 980, analyzing 200 ng of a HEK293 tryptic digest using a high-throughput 300 SPD method. We evaluated the DIA acquisition speed [Hz] as a function of IT on the Orbitrap Astral Zoom MS and compared it to the Orbitrap Astral MS (Fig. 2*A*). This analysis showed that the fastest scan rate of ∼270 Hz can be achieved on the Orbitrap Astral Zoom MS when IT is restricted to 1.5 ms or less, whereas the Orbitrap Astral MS can reach a maximum scan rate of ∼220 Hz when restricting the IT to 2.5 ms or less. Notably, when accounting for the 1 ms ‘free’ pre-Ac on the Orbitrap Astral Zoom MS, the effective optimal ion accumulation time of 1.5 ms aligns closely with the 2.5 ms optimal IT on the Orbitrap Astral MS, demonstrating consistency between the two instruments. As expected, the Orbitrap Astral Zoom MS outperformed the Orbitrap Astral MS instrument by at least 10% across all tested IT. The fastest scanning conditions also produced the most identifications with the Orbitrap Astral Zoom MS identifying ∼80,000 unique peptides compared to ∼67,000 peptides on the Orbitrap Astral MS, corresponding to a ∼20% increase in peptide identifications when operated under optimal conditions (Fig. 2*B*).

To systematically evaluate the individual contributions of pre-Ac, higher acquisition rate (HAR), and ESP in the new Orbitrap Astral Zoom MS, we independently benchmarked each feature in comparison to the Orbitrap Astral MS. Briefly, we performed a series of DIA experiments in which each feature was individually enabled using two distinct acquisition strategies: a high-sensitivity method (6 ms IT, 4 Th isolation windows, 50 SPD) and a fast-scanning method (1.5 ms IT, 2 Th windows, 90 SPD), using a range of peptide input amounts (20, 50, and 200 ng for the fast-scanning method; 50, 100, and 250 ng for the high-sensitivity method).

In the fast-scanning method, pre-Ac emerged as the primary contributor to the performance gains observed for the Orbitrap Astral Zoom MS (Fig. 2*C*, Supplemental Fig. S2 *A* and *B*). This was reflected in both the total number of unique peptides identified and the number of peptides quantified with a coefficient of variation < 20%, with pre-Ac alone accounting for the majority of the increase and achieving performance levels nearly equivalent to the Orbitrap Astral Zoom MS. This can be explained by the fast-scanning methods restricting the maximum IT to 1.5 ms, thereby limiting the number of ions that can be accumulated prior to analysis in the Astral analyzer (25). The resulting reduced ion population negatively affects spectral quality and sensitivity, particularly for low-abundance peptides. Consequently, the pre-Ac feature yields a higher number of peptide identifications at lower input amounts than Orbitrap Astral MS and other individual features, as at shorter ITs, pre-Ac enabled a relative increase in ion accumulation time of approximately 66%, resulting in higher average scan intensities and subsequent identification rate than those achieved by enabling either HAR or ESP alone (Supplemental Fig. S2*E*). In contrast, HAR or ESP features alone produced only modest gains in unique peptide identifications relative to the Orbitrap Astral MS, mainly at mid-to-high peptide loads, highlighting the advantage of combining all features in the Orbitrap Astral Zoom MS.

By contrast, when operating the instrument with a high-sensitivity method with slightly wider nDIA windows and longer ion ITs, the ESP algorithm contributed the largest performance gains (Fig. 2*D*, Supplemental Fig. S2, *C* and *D*). This shift in performance drivers relative to fast-scanning methods reflects the longer ion ITs allowed in the high-sensitivity regime, which reduce the relative contribution of scan overheads to total MS/MS cycle time. As a result, duty cycle efficiency and ion utilization are increased, diminishing the relative impact of the pre-Ac feature, which primarily exploits overhead time. Additionally, the increased transmission resulting from a wider DIA isolation window and longer ITs results in the acquisition of more feature-rich MS/MS spectra. These spectra are more effectively resolved when the ESP algorithm is enabled, which further facilitate better spectral matching in search engines, leading to an increased number of fragments per peptide matched, resulting in higher peptide search scores, and consequently higher identification rates (Supplemental Fig. S2*F*).

Overall, compared to the Orbitrap Astral MS, the combined advancements in both hardware and software within the Orbitrap Astral Zoom MS resulted in a 51% increase in the number of unique peptides identified when analyzing a 50 ng HEK293 tryptic digest. When considering individual features, performance improvements were 31% for pre-Ac, 38% for high acquisition rate, and 49% for enhanced spectral processing, relative to the performance of the Orbitrap Astral MS as shown in Supplemental Figure S2*G*. These results illustrate the synergistic effects of all features available with the Orbitrap Astral Zoom MS, enabling enhanced performance and identification rates when operating the instrument with different methods and sample loads.

### Evaluation of the Accuracy and Precision of LFQ on the Orbitrap Astral Zoom MS under Rapid Scanning Rates

To evaluate the quantitative performance of the Orbitrap Astral Zoom MS compared to the Orbitrap Astral MS in terms of precision and accuracy under high-speed scanning conditions (270 and 220 Hz respectively), we employed a label-free three-species proteome mixture, for which we mixed yeast and *E*. *coli* proteins in different ratios while keeping human proteins constant (Fig. 3*A*). The samples were acquired using a 50 SPD method with 2 Th isolation windows, and the maximum IT was set to 1.5 ms for both instruments.

Notably, across all conditions, the Orbitrap Astral Zoom MS consistently identified an average of ∼14,000 PGs, exceeding the Orbitrap Astral MS by > 1000 PG (Fig. 3*A*). Importantly, the quantitative accuracy of the Orbitrap Astral Zoom MS proved to be on par with or even exceeded the quantitative accuracy of the Orbitrap Astral MS based on the absolute median SDs of protein ratios compared to the theoretical ones (Fig. 3*B*). These results suggest that the faster scanning methods, which yield a higher number of PG identifications, do not have a negative impact on the quantitative performance.

To ensure a fair comparison, a second set of experiments was performed, where all instrument settings were kept the same except for the IT, which was set to 2.5 ms, representing the optimal IT for the Orbitrap Astral MS (Fig 2*B* and Supplemental Fig. S3*A*). As with the previous experiments, the Orbitrap Astral Zoom MS exhibited superior performance than the Orbitrap Astral MS. This improvement can be attributed to the higher scanning speed of the Orbitrap Astral Zoom MS, which allowed for the detection and quantification of more peptides than the Orbitrap Astral MS (Supplemental Fig. S3*B*). Furthermore, the increased scan speed of the Orbitrap Astral Zoom MS contributed to greater data completeness across all conditions (Supplemental Fig. S3*C*).

### Enhancing MS1 Dynamic Range via Multiplexed Ion Injections with Differential Accumulation Time Across m/z Segments

Although DIA has become the method of choice for single-shot proteomics due to its high sensitivity and reproducibility (Supplemental Fig. S4*A*), DDA remains valuable for specific applications, for example, the generation of spectral libraries (26), tandem mass tag–based experiments (27, 28, 29), and immunopeptidomics (30), among others. However, DDA is inherently constrained by the number of precursors that can be observed in full MS survey scans and subsequently selected for fragmentation in each acquisition scan cycle. When analyzing complex peptide mixtures, suppression of lower-abundance peptide species are frequently observed due to the restricted dynamic range of Orbitrap full MS1 scans, which is limited by the ion capacity of the C-trap (31). Consequently, when precursor ions are very low abundant, they may go undetected in full MS scans or fall below the threshold for MS/MS triggering, resulting in missed peptide identifications and reduced proteome coverage.

To overcome these constraints and expand both ion sampling and dynamic range in full-scan MS, we implemented the eDR mode on the Orbitrap Astral Zoom MS. In this mode, the MS1 mass range is partitioned into predefined wide selected ion monitoring windows, each acquired with differential ion ITs, and merges them into a single Orbitrap full scan, while MS/MS spectra are acquired in parallel using the Astral analyzer. To evaluate the performance of this mode, we performed a series of DDA experiments with a 100 SPD method benchmarking the Orbitrap Astral MS and the Orbitrap Astral Zoom MS with eDR mode disabled or enabled. As expected, although the Orbitrap Astral Zoom MS incorporates features such as pre-Ac and higher acquisition rates that improve ion utilization and scanning efficiency (data-dependent MS/MS scanning speed up to 200 Hz, Supplemental Fig. S4*B*), these enhancements cannot overcome DDA’s inherent limitation. As a result, only modest improvements in peptide and protein group identification rates were observed relative to the Orbitrap Astral MS. However, enabling the eDR mode on the Orbitrap Astral Zoom MS resulted in a substantial increase in precursor identifications, yielding ∼15,000 and ∼30,000 additional precursors compared to standard and wide-window DDA methods, respectively, compared to the Orbitrap Astral MS (Fig. 4*A*, Supplemental Fig. S4*C*). This improvement was a direct consequence of eDR MS1 consistently enabling the triggering ∼150 MS/MS scans per second, yielding >80k MS/MS spectra across the peptide elution window at a fixed MS2 IT of 3 ms. Conventional DDA methods operating without eDR were unable to sustain similarly high scan rates throughout the chromatographic gradient (Fig. 4*B*, Supplemental Fig. S4, *D* and *E*). This demonstrates that eDR-MS1 can help overcome the dynamic range issues in conventional full-scan MS and thereby increase the number of observable target precursor ions for triggering DDA-MS/MS spectra.

### Optimized Fractionation Strategy for Rapid and Comprehensive Human Proteome Profiling via Multi-Shot Proteomics

Based on next-generation sequencing data, human cancer cell lines like HeLa and HEK293 express ∼12,500 protein-encoding genes (8). We have previously demonstrated that using deep peptide fractionation via offline HpH reversed-phase chromatography in combination with online low-pH LC-MS/MS allows for the identification of essentially all the expressed proteins in a cell line (32). In the first implementation of this strategy, it took 34.5 h of MS measurement time to achieve near-complete proteome coverage, but with the Orbitrap Astral MS, this was reduced to just ∼4.5 h by analyzing 34 offline HpH fractions of a tryptic digest of HEK293 cells with a 180 SPD nDIA (8). This represented a 7x improvement in throughput, enabling deep proteome profiling with dramatically increased efficiency. To test whether near-complete human proteome coverage could be achieved in even shorter analytical timeframes using the Orbitrap Astral Zoom MS, we fractionated a HEK293 peptide digest by offline HpH into 24, 34, and 46 fractions. Each fractionation scheme was analyzed using 180 or 300 SPD method (Fig. 5*A*).

We investigated the proteome coverage achieved for each fractionation scheme as a function of MS measurement time and compared the results to the 1-h single-shot analysis (24 SPD) and the 34 fractions data published previously. (Fig. 5*B*). Using a 1 h gradient, we achieved a single-shot proteome coverage of 10,247 proteins; approximately 2000 proteins short of a complete proteome. In contrast, by analyzing 34 HpH fractions by nDIA with 300 SPD LC method, the Orbitrap Astral Zoom MS significantly expanded the proteome coverage while using just ∼3 h of total LC-MS analysis time. This enabled the identification of more than 12 thousand protein groups, with the majority of those uniquely detected by the multifraction analysis corresponding to low-abundant proteins, including membrane proteins and transcription factors (Supplemental Fig. S5*A*). This represented close to two-fold increase in throughput for comprehensive proteome profiling compared to the Orbitrap Astral MS and a 14 × increased throughput compared to the Q Exactive HF-X MS (32).

We identified a comparable number of proteins using the faster 300 SPD analysis, although the >200,000 unique peptides identified were ∼10% fewer than the 180 SPD method, likely reflecting the reduced chromatographic resolution inherent to shorter gradients (Fig. 5*B*). For both 180 and 300 SPD analyses, we achieved very high overall protein sequence coverage, which also enables analysis of major post-translational modifications without specific enrichment. For instance, searching the 34 fractions for phosphorylated peptides allowed identification of 2089 phosphorylation sites, which adhered to the expected target amino acid residues (33), with ∼85% serine, ∼15% threonine, and <1% tyrosine modification (Fig. 5*C*). Sequence logo motif analysis and molecular function gene ontology (GO) term enrichment revealed a strong enrichment for substrates of proline-directed kinases, such as cyclin-dependent kinases, which play critical roles in regulating cell cycle progression and modulating processes related to DNA replication and checkpoint control (34, 35) (Supplemental Fig. S5, *B* and *C*). Importantly, since we analyzed protein phosphorylation from nonenriched samples analyzed with very short LC gradients, we estimated false localization rates by performing a stringent decoy strategy wherein we additionally considered alanine residues as a mock phosphorylation target. At high site localization probabilities (>90%), we observed an estimated false localization rate of ∼4%, highlighting an overall robust identification of phosphopeptides and site localization assignment (Supplemental Fig. S6).

Furthermore, the near-complete HEK293 proteome data also allowed us to identify protein variants, including alternative splicing events, SAVs, and splicing-associated variants including insertions and deletions (indels) by searching the data against a large protein database that was translated from transcriptomic information about HEK293 variants. In total, we identified 935 alternative splicing events, 743 SAVs, and 11 indels (Fig. 5*D*). To ensure strict control of false discovery rate, we investigated the distribution of target and decoy hits specifically for SAVs and compared them to all other peptides (Supplemental Fig. S7). This analysis showed no difference in target-decoy distributions, indicating consistent FDR control and the robustness of the identified SAVs. GO analysis of the detected splicing variants revealed enrichment in biological processes related to DNA repair and embryonic development, consistent with regulatory functions during cell proliferation and genome maintenance. These findings reflect the central role of alternative splicing as a key mechanism for expanding proteomic diversity, particularly within genes involved in regulatory, signaling, and developmental pathways (36). By contrast, SAVs were predominantly enriched in mitochondrial and metabolic pathways, including the carnitine shuttle, mitochondrial electron transport chain, and ciliary body morphogenesis, which can reflect genetic variation or adaptation in long-term cultured cells, particularly impacting energy-producing organelles such as mitochondria (37) (Supplemental Fig. S5, *D* and *E*).

### Fast and Deep Proteome Profiling with Orbitrap Astral Zoom MS and Short-Gradient LC

To fully leverage the maximum acquisition speed of 270 Hz in nDIA mode of the Orbitrap Astral Zoom MS, we used the Vanquish Neo UHPLC in trap-and-elute mode, facilitating 300 SPD analysis. Analyzing 200 ng of HEK293 tryptic digest with the fastest nDIA method using 2 Th isolation windows and 1.5 ms maximum IT, we identified ∼100,000 unique peptides and 8400 protein groups (Fig. 6*A*), with excellent quantitative reproducibility across technical replicates (Pearson correlation coefficient of 0.99 for >8400 proteins; Fig. 6*B*).

To evaluate whether even higher-throughput proteome analysis is feasible, we employed the Evosep Eno LC, which provides enhanced synchronization of valve switching and pump control. This improvement enables robust chromatographic performance, particularly under ultra-fast gradient conditions such as the 500 SPD method, making it suitable for large-scale proteomic studies involving thousands of samples, including clinical research and systems biology applications. The 500 SPD method is designed for ultra-high throughput using a 2.2 min gradient with a total run-to-run time of only 2.88 min. Analyzing 200 ng of HEK293 tryptic digest with the 500 SPD method, we found that the Orbitrap Astral Zoom MS identified on average 7136 unique protein groups from ∼67,000 unique peptides, which is > 15,000 more peptides than covered by the Orbitrap Astral MS (Fig. 6*C*). This was achieved with very high reproducibility between replicates with Pearson correlation coefficient of >0.99, consistently quantifying >7000 proteins between replica runs (Fig. 6*D*). The 500 SPD method provided 72 s of active peptide elution across the gradient, and during the main part of this, >1000 unique peptides per second were identified on average when using the Orbitrap Astral Zoom MS (Supplemental Fig. S8*A*). The cumulative number of unique peptides identified from the onset of the active gradient increased linearly, with 50,000 unique peptides identified within the first 50 s (Supplemental Fig. S8*B*). As peptides from high-abundant proteins are more easily identified, we did not expect to see a similar linear increase in unique proteins across the gradient. Indeed, the cumulative number of unique proteins initially grew very fast with 2000 proteins identified in the first 7 s, 4000 proteins identified in the first 14 s, and 6000 proteins identified in the first 30 s of active gradient, ultimately reaching saturation with ∼7000 proteins identified after approximately 1 minute (Supplemental Fig. S8*C*).

To benchmark LFQ accuracy and precision under fast LC conditions, we applied the three-species hybrid strategy with two very rapid LC gradients (500 SPD and 300 SPD) across three different nDIA MS acquisition schemes. We compared a reference method (2 Th isolation windows, 1.5 ms IT, m/z 380–980) with two alternative methods designed to probe the effects of wider isolation windows, narrower m/z ranges, and shorter overall nDIA scan cycle time: method 1 (4 Th isolation windows, 2 ms IT, m/z 400–800) and method 2 (4 Th isolation windows, 2 ms IT, m/z 400–600). This systematic comparison allowed us to assess how acquisition parameters influence quantification performance under high-throughput conditions. The reference method yielded the highest number of peptide (∼81,200 and ∼58,000) and protein group (∼9600 and ∼8300) identifications in the 300 SPD and 500 SPD gradients, respectively (Supplemental Fig. S9, *A*–*B*). It showed acceptable SDs and log_2_ error deviations for high- and medium-abundance ratios but exhibited greater variability for low-abundance ratios (Supplemental Figs. S10–12). A closer evaluation of quantification accuracy using species-specific SDs showed that method 2 achieved the best quantitative precision, despite lower identifications. By contrast, method 1 provided the best balance between proteome depth (∼9000 and ∼7200 PG identifications in the 300 SPD and 500 SPD gradients, respectively) and quantitative accuracy (Supplemental Figs. S11,12).

These differences can be explained by the number of data points per peak (DPPP) across gradients. In the reference method, most proteins are supported by sufficient peptide measurements with acceptable DPPP for high- and medium-abundance ratios, allowing accurate ratio estimation (Supplemental Fig. S13). In contrast, for low-abundance ratios, many peptides exhibit limited DPPP, causing larger deviations from the expected target ratios (Supplemental Fig. S13). In 300 SPD DIA runs (method 1), the median chromatographic peak full width at half maximum (FWHM) of 1.56 s combined with an MS2 cycle time of 397 ms yielded a median DPPP of 5 (IQR = 3) (Supplemental Fig. S14*A*). Conversely, in 500 SPD runs (method 1), the median FWHM of 1.35 s and MS2 cycle time of 424 ms resulted in a lower median DPPP of 3 (IQR = 1) (Supplemental Fig. S14*B*). Together, these results indicate that our ultra-short-gradient methods maintain sufficient sampling density to support robust and accurate quantification.

Finally, we performed a long-duration stability test using the 300-SPD and 500-SPD scanned with nDIA method 1 and reference method, respectively, injecting 200 ng per run for more than 24 h (equivalent to 550 and 321 samples, respectively). Reassuringly, the columns used for each method demonstrated stability, with median FWHM values of 0.022 min (300-SPD) and 0.032 min (500-SPD). MS1 signal intensity remained consistent across all injections and gradients. In addition, identification performance was stable throughout the entire sequence, with median identification rates of 8422 protein groups for the 300-SPD method and 7867 protein groups for the 500-SPD method (Supplemental Fig. S15). Together, these results indicate that our ultra-short-gradient methods maintain stability and sufficient sampling density to support robust and accurate quantification.

### A Comprehensive Draft of the Human Proteome Atlas via Ultra-Fast LC-MS

**Fig. 7:**
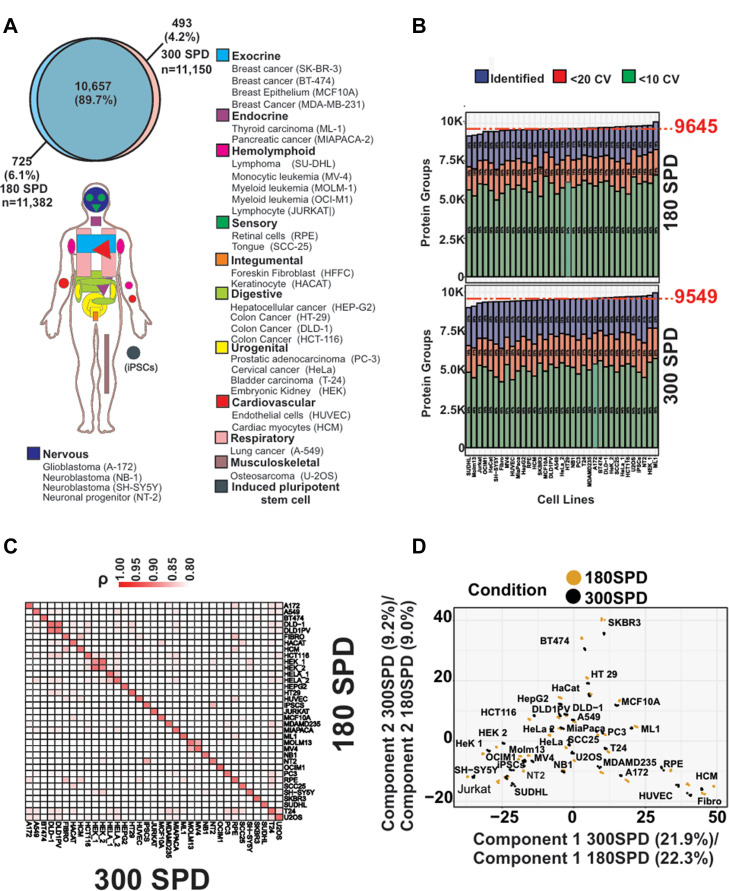
**Fast and deep proteome profiling with the Orbitrap Astral Zoom MS using short-gradient LC**. *A*, overview of the tissue origins of 32 human cell lines profiled in technical quadruplicates using DIA with 300 SPD and 180 SPD LC-MS/MS workflows (*bottom*). Venn diagram (*top*) shows the overlap and total number of protein groups identified across both gradient conditions. *B*, bar plot of the protein groups detected at 300 SPD (*top*) and 180 SPD (*bottom*). Median identifications are indicated by a *red dashed* line. Coefficient of variation (CV) below 10% and 20% CV (n = 4) are depicted in *green* and *red*, respectively. *C*, Pearson correlation heatmap of the 180 SPD and 300 SPD datasets. *D*, PCA analysis of the 32 cell lines recorded at 180 SPD and 300 SPD. (Data processed with SN v19).

To assess coverage and quantitative performance of fast LC gradients for human proteome profiling, we analyzed a panel of 32 human cell lines representing cell types of the major organs. All samples were analyzed with both 180 SPD and 300 SPD in quadruplicates with the Orbitrap Astral Zoom MS, and collectively, we identified >11,500 proteins with high overall reproducibility between the two LC gradients (Fig. 7*A*).

Surprisingly, we found that the average protein coverage per cell line was comparable between the 180 SPD and 300 SPD with an average 9645 proteins and 9549 proteins identified, respectively (Fig. 7*B*). Reassuringly, we found very high correlations between cell line proteomes representing the same cell type between the 180 SPD and 300 SPD (Fig. 7*C*). Moreover, principal component analysis of the cell line proteomes acquired with 180 SPD and 300 SPD gradients were almost perfectly overlayed when visualizing the two major principal components, indicating high concordance in global proteomic profiles across the two acquisition methods (Fig. 7*D*). The Euclidean distance between cell lines was preserved across gradients, indicating consistent relative proteomic relationships within each acquisition method. The GO analysis of different clusters derived from hierarchical heat map clustering confirmed consistent enrichment of biological processes across both 180 SPD and 300 SPD workflows, highlighting the consistency of the proteomic signal across gradient lengths. (Supplemental Fig. S16)

## Discussion

The development of the Orbitrap Astral Zoom mass spectrometer represents a significant advancement in proteomics instrumentation. By achieving ultra-fast MS/MS scan rates while maintaining high sensitivity, quantitative accuracy, and proteome depth, this mass spectrometer overcomes longstanding limitations in scalability and speed. In combination with very fast online LC gradients, this platform has the potential to redefine the landscape of proteomics research, enabling more comprehensive and efficient exploration of biological systems at an unprecedented throughput and scale.

Building upon the Orbitrap Astral MS technology, the Orbitrap Astral Zoom MS introduces key advancements in duty cycle efficiency, ion-accumulation, ion-transfer, and spectral processing. These modifications result in a ∼23% increase in MS/MS acquisition speed, rising to 270 Hz compared to the maximum of ∼220 Hz of the Orbitrap Astral MS. The increased scan rate is accompanied by sufficient sensitivity and quantitative reproducibility to bridge the gap between DDA and DIA methods. These advancements extend previously established Orbitrap Astral MS capabilities, by enabling faster analysis times while maintaining, or even improving, proteome coverage and quantitative reproducibility.

The Orbitrap Astral Zoom mass spectrometer allows for near-complete proteome coverage within a fraction of the acquisition time required by earlier instruments. This is exemplified by the identification of >12,000 proteins in only 2.7 h via offline HpH reversed-phase peptide chromatography combined with very fast online 300 SPD LC-MS/MS analysis. Furthermore, the ability to analyze up to 500 SPD with high reproducibility (Pearson correlation coefficients >0.99) opens doors for large-scale biomarker discovery, systems biology, and high-throughput drug screening. This substantial reduction in acquisition time and cost makes comprehensive proteomics more accessible, expanding its adoption makes comprehensive proteomics more accessible across diverse research fields. By dramatically increasing throughput without sacrificing data quality, the Orbitrap Astral Zoom MS enables large-scale, high-resolution proteomics studies that were previously impractical or prohibitively expensive. Applications in longitudinal studies, multiomics integration, and single-cell proteomics are now more feasible. Additionally, the improved precision and reproducibility of the instrument enhance the reliability of datasets, making them more suitable for advanced statistical modeling and machine learning approaches, which often require high-quality, large-scale data. Thus, the Orbitrap Astral Zoom MS sets the precedent for future developments, such as integrating proteomics with other omics platforms and advancing real-time proteome monitoring in clinical settings.

The technological innovations in the Orbitrap Astral Zoom MS, in particular the parallel ion pre-Ac, faster quadrupole ion transfer, and enhanced spectral processing algorithms, are central to its performance. These features complement each other, with pre-Ac and faster ion transfer adding 66% effective IT, facilitating an extra 70 scans per second at the same sensitivity. Furthermore, the efficacy of enhanced spectral processing enables resolving and interpretation of the more complex spectra generated when analyzing highly complex samples with slower and more sensitive methodology. Collectively, these features ensure increased performance regardless of acquisition method, enabling the instrument to achieve both increased scan rates and greater proteome depth while maintaining high sensitivity. The introduction of eDR mode further expands its utility, enabling deeper peptide and protein identification with DDA. This technological leap ensures that the Orbitrap Astral Zoom MS is well-suited for both exploratory and targeted proteomics applications.

As we demonstrated in this study by analyzing 300 or 500 SPD with high reproducibility, the Orbitrap Astral Zoom MS enables rapid profiling of human cell lines and clinical research samples. The capability for deep and high-throughput analysis is critical for studying heterogeneous biological systems, such as cancer tissues or immune responses, where large sample sizes are required to capture variability and moreover supports the development of personalized medicine approaches by enabling the rapid identification of disease-specific biomarkers.

While the instrument represents a significant advancement, certain limitations remain. For example, the ultra-short LC gradients (e.g., 300 and 500 SPD) may not detect low-abundant proteins such as cell type–specific transcription factors in highly complex samples and, although partially compensated for by higher scan rates, very short chromatographic gradients inherently limit precursor peak width, potentially inhibiting precise quantification. However, we demonstrate that even using ultra-short LC gradients, acceptable quantitative performance can be retained when using suitable LC-MS methodology (Supplemental Figs. S11 and S12). Finally, with the Orbitrap Astral Zoom MS achieving remarkable scan rates and data density, the effective utilization of this capability depends on downstream data analysis pipelines that can handle the increased data volume.

In the context of ultra-low input (picogram range) analysis including single-cell proteomics, we recently demonstrated that the Orbitrap Astral Zoom MS provides 10 to 30% gains in PGs compared to the Orbitrap Astral MS (38). Besides this, several questions remain unanswered, such as how the Orbitrap Astral Zoom MS performs in more diverse sample types, beyond standard cell lines and model organisms. Furthermore, its ability to resolve post-translational modifications with ultra-fast scanning methods remains to be fully explored. Finally, while the instrument has shown excellent reproducibility, its performance in longitudinal studies with highly dynamic proteomes warrants further investigation.

In summary, the Orbitrap Astral Zoom mass spectrometer represents a significant landmark advancement in proteomics technology, combining high speed, sensitivity, and scalability. It sets a new standard for large-scale biological and clinical research, with the potential to profoundly impact on medical research, particularly in the areas of biomarker discovery, disease stratification, and therapeutic monitoring. The ability to analyze large clinical research cohorts rapidly and with high reproducibility will facilitate the identification of disease-associated proteomic signatures, paving the way for real-time monitoring of patient proteomes. In the long term, this technology could support the development of personalized medicine approaches by providing detailed, patient-specific proteomic insights. While challenges remain, the instrument sets the stage for the next generation of proteomics research, enabling unprecedented insights into the complexity of biological systems.

## Inclusion and Ethics Statement

We are committed to promoting diversity and inclusion in science and ensuring that our research is conducted ethically and responsibly. Our study was designed and conducted in accordance with ethical principles and guidelines, including obtaining informed consent from all participants and complying with relevant regulations and laws.

## Data Availability

Data are available via the ProteomeXchange with the following identifiers: (1) Features titration (90 SPD): PXD066925; Features titration (50SPD-250 ng):PXD067363, Features titration (50SPD-100 ng): PXD067317, Features titration (50SPD-50 ng): PXD067433; (2) Injection time titration (Astral Zoom): PXD066924; (3) Injection time titration (Astral): PXD066927; (4) DDA and DIA: PXD066944; (5) three-species mix: PXD066672; (6) 300 to 500 SPD: PXD066718 and PXD071972; (7) HpH fractionation schemes: PXD066659; and (8) cell lines: PXD066686. Supplemental Table S2 contains an overview of the experiments. Source data are provided with this paper.

## Supplemental Data

This article contains supplemental data.

## Conflict of Interest

The Olsen laboratory at the University of Copenhagen has a sponsored research agreement with Thermo Fisher Scientific, the manufacturer of the instrumentation used in this research. However, analytical techniques were selected and performed independent of Thermo Fisher Scientific. H. S., E. Denisov, B. H., J. P, A. K., Y. M, T. N. A., I. C., J. K., K. L. F., E. C., J-P. H, D. H, V. Z., C. H, and E. Damoc, are employees of Thermo Fisher Scientific, manufacturer of instrumentation used in this work. Thermo Fisher Scientific provides support to J. V. O.’s laboratory under a confidentiality agreement with the Novo Nordisk Foundation Center for Protein Research, University of Copenhagen. J. V. O., U. H. G., I. A. H., M. R., C. K. and O. Ø. are employees of the University of Copenhagen and declare no further competing interests.

## References

[1] Wang Z., Mülleder M., Batruch I., Chelur A., Textoris-Taube K., Schwecke T. (2022). High-throughput proteomics of nanogram-scale samples with Zeno SWATH MS. Elife.

[2] Messner C.B., Demichev V., Muenzner J., Aulakh S.K., Barthel N., Röhl A. (2023). The proteomic landscape of genome-wide genetic perturbations. Cell.

[3] Poulos R.C., Hains P.G., Shah R., Lucas N., Xavier D., Manda S.S. (2020). Strategies to enable large-scale proteomics for reproducible research. Nat. Commun..

[4] Bader J.M., Albrecht V., Mann M. (2023). MS-Based proteomics of body fluids: the end of the beginning. Mol. Cell Proteomics.

[5] Meier F., Brunner A.D., Frank M., Ha A., Bludau I., Voytik E. (2020). diaPASEF: parallel accumulation–serial fragmentation combined with data-independent acquisition. Nat. Methods.

[6] Skowronek P., Krohs F., Lubeck M., Wallmann G., Itang E.C.M., Koval P. (2023). Synchro-PASEF allows precursor-specific fragment ion extraction and interference removal in data-independent acquisition. Mol. Cell Proteomics.

[7] Messner C.B., Demichev V., Bloomfield N., Yu J.S.L., White M., Kreidl M. (2021). Ultra-fast proteomics with scanning SWATH. Nat. Biotechnol..

[8] Guzman U.H., Martinez-Val A., Ye Z., Damoc E., Arrey T.N., Pashkova A. (2024). Ultra-fast label-free quantification and comprehensive proteome coverage with narrow-window data-independent acquisition. Nat. Biotechnol..

[9] Torun F.M., Virreira Winter S., Doll S., Riese F.M., Vorobyev A., Mueller-Reif J.B. (2023). Transparent exploration of machine learning for biomarker discovery from proteomics and omics data. J. Proteome Res..

[10] Mann M., Kumar C., Zeng W.F., Strauss M.T. (2021). Artificial intelligence for proteomics and biomarker discovery. Cell Syst.

[11] Michalski A., Cox J., Mann M. (2011). More than 100,000 detectable peptide species elute in single shotgun proteomics runs but the majority is inaccessible to data-dependent LC-MS/MS. J. Proteome Res..

[12] Peters-Clarke T.M., Coon J.J., Riley N.M. (2024). Instrumentation at the leading edge of proteomics. Anal Chem..

[13] Stewart H.I., Grinfeld D., Giannakopulos A., Petzoldt J., Shanley T., Garland M. (2023). Parallelized acquisition of orbitrap and astral analyzers enables high-throughput quantitative analysis. Anal Chem..

[14] Grinfeld D., Stewart H., Balschun W., Skoblin M., Hock C., Makarov A. (2024). Multi-reflection astral mass spectrometer with isochronous drift in elongated ion mirrors. Nucl. Instrum Methods Phys. Res. A..

[15] Stewart H., Grinfeld D., Wagner A., Kholomeev A., Biel M., Giannakopulos A. (2024). A conjoined rectilinear collision cell and pulsed extraction ion trap with auxiliary DC electrodes. J. Am. Soc. Mass Spectrom..

[16] Hagedorn B., Grinfeld D., Dwivedi A., Stewart H. (2023).

[17] Batth T.S., Tollenaere M.A.X., Rüther P., Gonzalez-Franquesa A., Prabhakar B.S., Bekker-Jensen S. (2019). Protein aggregation capture on microparticles enables multipurpose proteomics sample preparation. Mol. Cell Proteomics.

[18] Demichev V., Messner C.B., Vernardis S.I., Lilley K.S., Ralser M. (2020). DIA-NN: neural networks and interference correction enable deep proteome coverage in high throughput. Nat. Methods.

[19] Pham T.V., Henneman A.A., Jimenez C.R. (2020). Iq: an R package to estimate relative protein abundances from ion quantification in DIA-MS-based proteomics. Bioinformatics.

[20] Bekker-Jensen D.B., Bernhardt O.M., Hogrebe A., Martinez-Val A., Verbeke L., Gandhi T. (2020). Rapid and site-specific deep phosphoproteome profiling by data-independent acquisition without the need for spectral libraries. Nat. Commun..

[21] Wu T., Hu E., Xu S., Chen M., Guo P., Dai Z. (2021). clusterProfiler 4.0: a universal enrichment tool for interpreting omics data. Innovation.

[22] Chambers M.C., Jagtap P.D., Johnson J.E., McGowan T., Kumar P., Onsongo G. (2017). An accessible proteogenomics informatics resource for cancer researchers. Cancer Res..

[23] Malm M., Saghaleyni R., Lundqvist M., Giudici M., Chotteau V., Field R. (2020). Evolution from adherent to suspension: systems biology of HEK293 cell line development. Sci. Rep..

[24] Grinfeld D. (2014). 2015 (30) foreign application priority data sep. Time Flight Mass Spectrometer. Anal. Chem..

[25] Harking F., Guzman U.H., Kraegenbring J., Stewart H., Aizikov K., Koch H. (2025). Enhancing tandem MS sensitivity and peptide identification via ion pre-accumulation in an orbitrap mass spectrometer. J. Proteome Res..

[26] Willems P., Fels U., Staes A., Gevaert K., Van Damme P. (2021). Use of hybrid data-dependent and -Independent acquisition spectral libraries empowers dual-proteome profiling. J. Proteome Res..

[27] Li J., Cai Z., Bomgarden R.D., Pike I., Kuhn K., Rogers J.C. (2021). TMTpro-18plex: the expanded and complete set of TMTpro reagents for sample multiplexing. J. Proteome Res..

[28] He Y., Yang K., Li S., Zeller M., McAlister G.C., Stewart H.I. (2025). TMT-based multiplexed (Chemo)Proteomics on the orbitrap astral mass spectrometer. Mol. Cell Proteomics.

[29] Ghosh G., Shannon A.E., Searle B.C. (2024). Data acquisition approaches for single cell proteomics. Proteomics.

[30] Leddy O., Cui Y., Ahn R., Stopfer L., Choe E., Kim D.H. (2024). Validation and quantification of peptide antigens presented on MHCs using SureQuant. Nat. Protoc..

[31] Meier F., Geyer P.E., Virreira Winter S., Cox J., Mann M. (2018). BoxCar acquisition method enables single-shot proteomics at a depth of 10,000 proteins in 100 minutes. Nat. Methods.

[32] Bekker-Jensen D.B., Kelstrup C.D., Batth T.S., Larsen S.C., Haldrup C., Bramsen J.B. (2017). An optimized shotgun strategy for the rapid generation of comprehensive human proteomes. Cell Syst..

[33] Olsen J.V., Blagoev B., Gnad F., Macek B., Kumar C., Mortensen P., Mann M. (2006). Global, in vivo, and site-specific phosphorylation dynamics in signaling networks. Cell.

[34] Dephoure N., Zhou C., Villén J., Beausoleil S.A., Bakalarski C.E., Elledge S.J., Gygi S.P. (2008). A quantitative atlas of mitotic phosphorylation. Proc. Natl Acad. Sci. USA.

[35] Daub H., Olsen J.V., Bairlein M., Gnad F., Oppermann F.S., Körner R. (2008). Kinase-selective enrichment enables quantitative phosphoproteomics of the kinome across the cell cycle. Mol. Cell.

[36] Nilsen T.W., Graveley B.R. (2010). Expansion of the eukaryotic proteome by alternative splicing. Nature.

[37] Granath-Panelo M., Kajimura S. (2024). Mitochondrial heterogeneity and adaptations to cellular needs. Nat. Cell Biol..

[38] Hendriks I.A., Buch-Larsen S.C., Rykær M., Lechner M.Y., Kverneland A.H., Arrey T.N. (2025). From HeLa to human blood: extending single-cell proteomics to the smallest immune cells. bioRxiv.

