# Supplementary material for: Higher-Throughput Proteome Profiling Enabled by Parallelized Pre-Accumulation and Optimized Ion Processing in the Orbitrap Astral Zoom Mass Spectrometer
Source: Mol Cell Proteomics. 2026 Jan 9;25(2):101504. doi: 10.1016/j.mcpro.2025.101504 (PMC12914409; doi:10.1016/j.mcpro.2025.101504)
Supplement: Supplemental — Material [file mmc1.docx]

**Supplementary Materials**


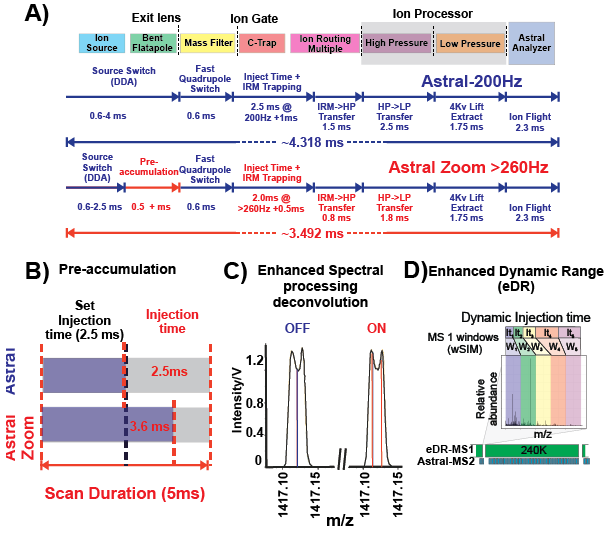


**Supplementary Fig. 1.** **Schematics of the Orbitrap Astral Zoom Mass Spectrometer`s Ion Processing Features. A)** Ion processing scheme depicting the improvements made to parallelized stages and timings of the Orbitrap Astral Zoom MS compared to the Orbitrap Astral MS. Median scan-scan duration is depicted for both instruments. **B)** Scheme illustrating the Pre-Accumulation feature concept when the injection time is set to 2.5 ms and no HAR enabled. **C)** Profile spectrum of a split ion peak with centroid positions when the Enhanced Spectral deconvolution algorithm (ESP) is enabled or disabled. **D)** Schematic illustrating the enhanced dynamic range (eDR) mode, featuring multiplexed ion injections with distinct accumulation times tailored to specific m/z ranges.

**
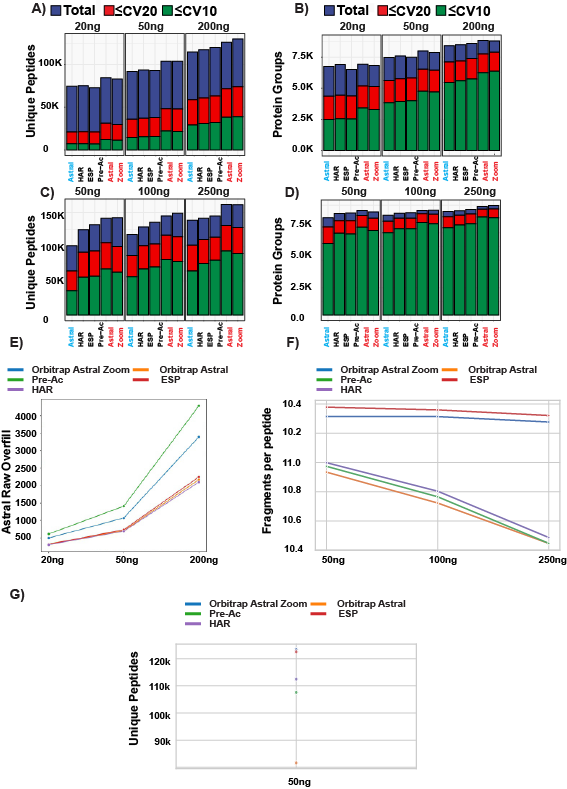
**

**Supplementary Fig. 2.** **Performance Benchmarking of the Orbitrap Astral Zoom MS Features. A,C)** Number of unique peptides and **B,D)** number of protein groups identified across a dilution series of HEK293 peptides (20–200 ng or 20–250 ng) using 90 SPD and 50 SPD gradients. The upper panels represent data with 2 Th isolation windows and 1.5 ms injection time, while the lower panels show results with 4 Th isolation windows and 6.0 ms injection time. The performance of individual features enabled on the Orbitrap Astral Zoom MS is compared to that of the Orbitrap Astral MS. Coefficients of variation (CV) below 10% and 20% across five replicates (n = 5) are indicated in green and red, respectively. Feature abbreviations: HAR, high acquisition rate; Pre-Ac, pre-accumulation; ESP, Enhanced spectral processing. **E)** Average scan-level ion overfill observed during 90 SPD acquisitions across 20-200 ng tryptic peptides from HEK293 with individual features enabled on the Orbitrap Astral Zoom MS and Orbitrap Astral MS. **F)** Average number of fragment ions per peptide identified across a dilution series (50–250 ng) of HEK293 tryptic peptides with individual features enabled during 50 SPD LC gradients (4 Th isolation windows, 6.0 ms injection time), compared to Orbitrap Astral Zoom MS and Orbitrap Astral MS. **G)** Unique identified peptides count from 50 ng HEK293 tryptic peptide injections with individual features enabled during 50 SPD LC gradients (4 Th isolation windows, 6.0 ms injection time), benchmarked against the Orbitrap Astral MS and Orbitrap Astral Zoom MS.

**
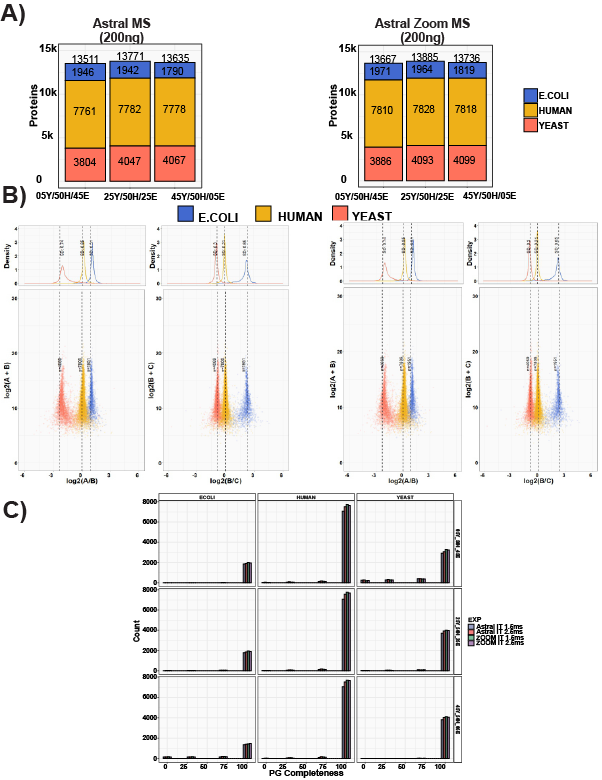
**

**Supplementary Fig. 3.** **Quantitative Assessment of LFQ Accuracy and Precision on the Orbitrap Astral Zoom MS under High-Speed Scanning Regimes (2.5 ms).** Samples were processed using the Orbitrap Astral MS and Orbitrap Astral Zoom MS in technical triplicates, employing a 2.5-ms maxIT and 2-Th window size method. The loading amounts were 200 ng. **A)** Number of proteins identified from the three species in each sample. For the Orbitrap Astral MS and Orbitrap Astral Zoom MS. **B)** log-transformed ratios of quantified proteins. Scatter plots for all runs over the log-transformed protein intensities are displayed at the bottom, while density plots are on the top. Colored dashed lines represent expected log2(A/B) values for proteins from humans (yellow), yeast (orange) and E. coli (blue). Standard deviations are displayed on the density plots. **C)** Data completeness is depicted for each ratio and organism.

**
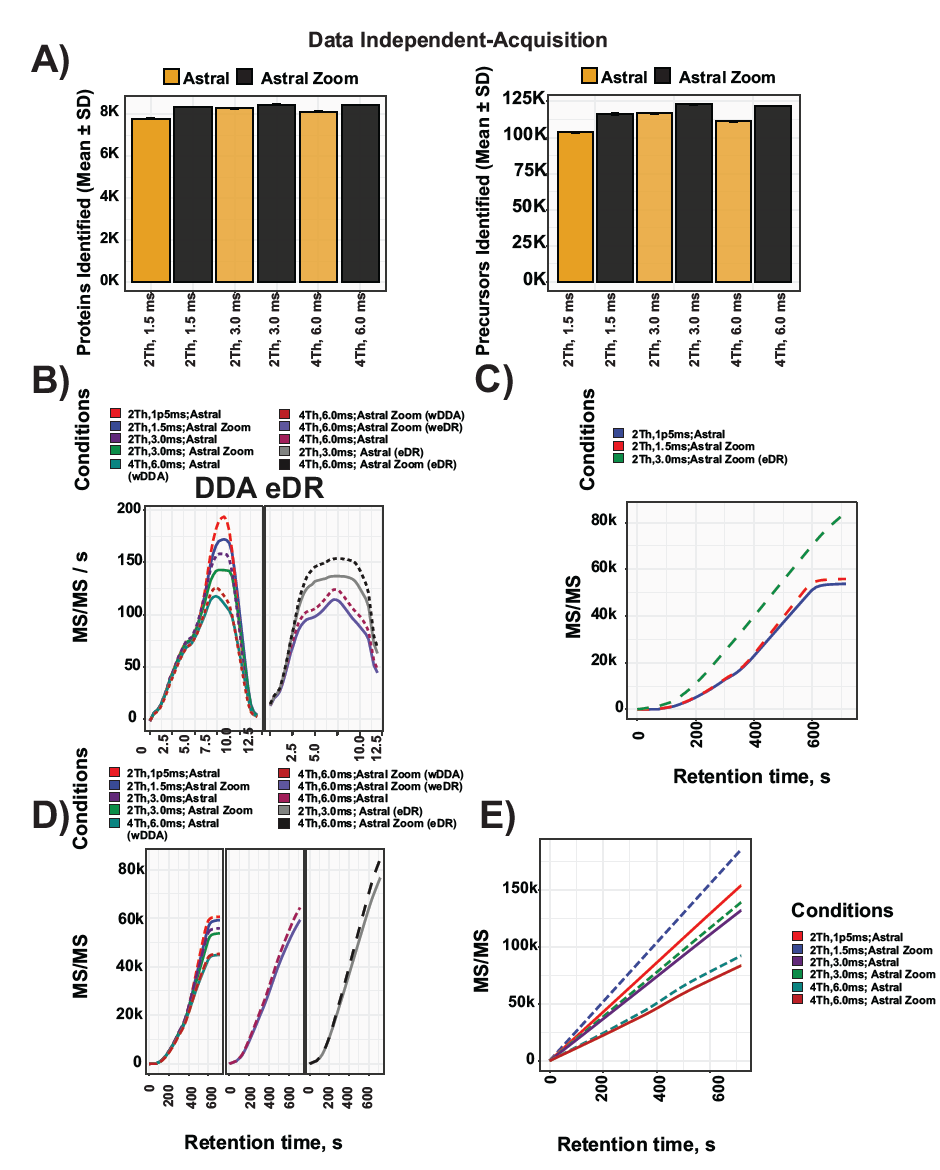
**

**Supplementary Fig. 4.** **Performance benchmarking of the Orbitrap Astral Zoom MS in DDA and DIA acquisition modes. A)** Data-Independent acquisition (DIA) performance at the protein group and peptide levels comparing the Orbitrap Astral Zoom MS to Orbitrap Astral MS. Analyses were performed at 100 SPD using 10 ng HEK 293 tryptic peptide input across four distinct DIA methods. **B)** MS/MS acquisition rate (scans per second) over the LC gradient for various DDA methods. **C-D)** Cumulative MS/MS scan counts as a function of retention time, comparing DDA methods on the Orbitrap Astral and the Orbitrap Astral Zoom MS with eDR mode on and off. **E)** Cumulative MS/MS scan counts over LC retention time for various DIA acquisition strategies, comparing fast- and high-sensitive methods across the Orbitrap Astral MS and the Orbitrap Astral Zoom MS.


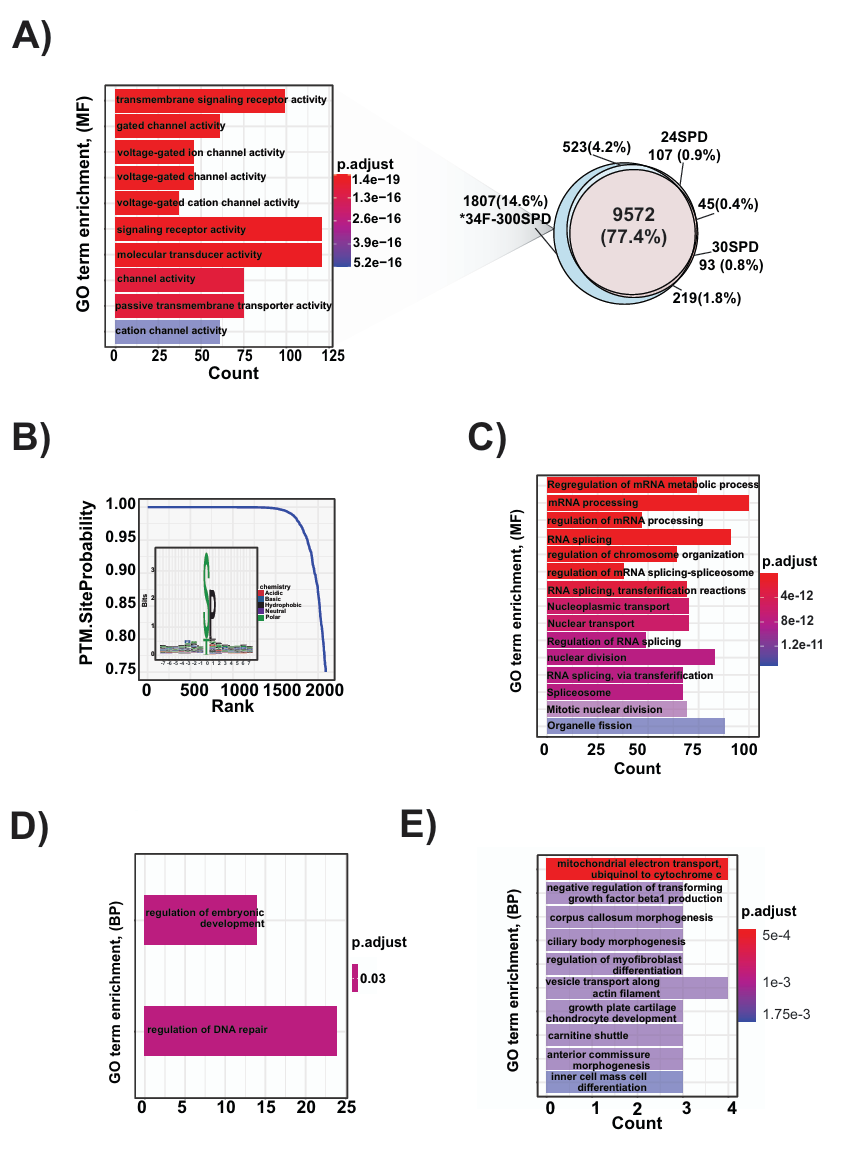


**Supplementary Fig. 5. Multi-shot proteomics strategy enables detection of low-abundance peptides, post-translational modifications, and protein variants. A)** Gene Ontology (GO) enrichment analysis of *Molecular Function* terms for proteins uniquely identified using the multi-shot approach compared to single-shot acquisition at 24 SPD.
**B)** GO enrichment analysis of *Molecular Function* terms for phosphorylated proteins identified without enrichment using 34 high-pH reversed-phase (HpH) fractions acquired at 300 SPD. **D-E)** GO enrichment analysis of *Biological Process* terms associated with splice variants (**D**) and single amino acid variants (SAVs; **E**) detected using the multi-shot proteomics strategy with 34 HpH fractions.


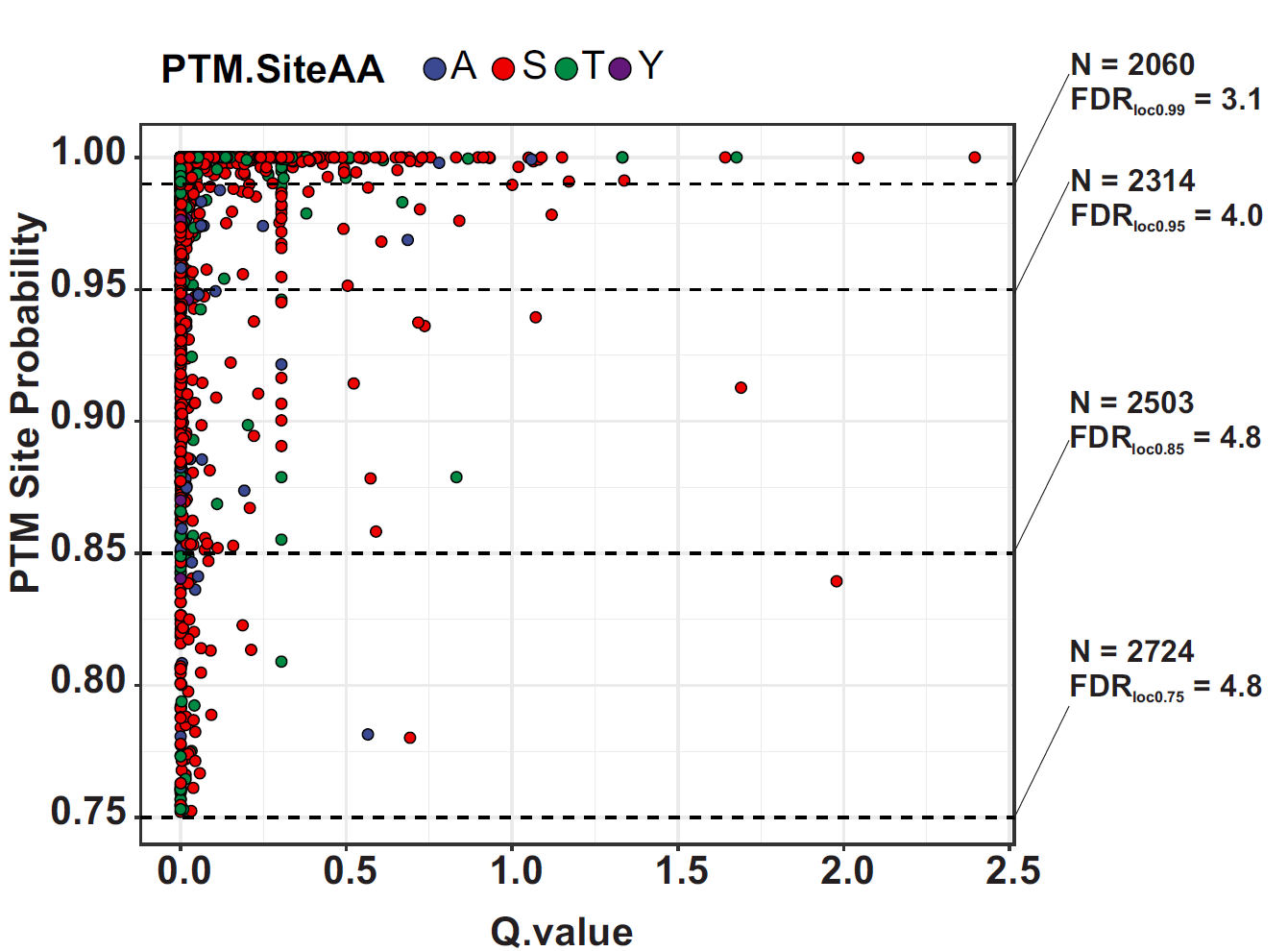


**Supplementary Fig. 6. PTM site probability as a function of the Q-value determined by SN v.19.** Empirical localization FDR was estimated using alanine phosphorylation as an entrapment strategy to assess FDR at different PTM localization probabilities**.**


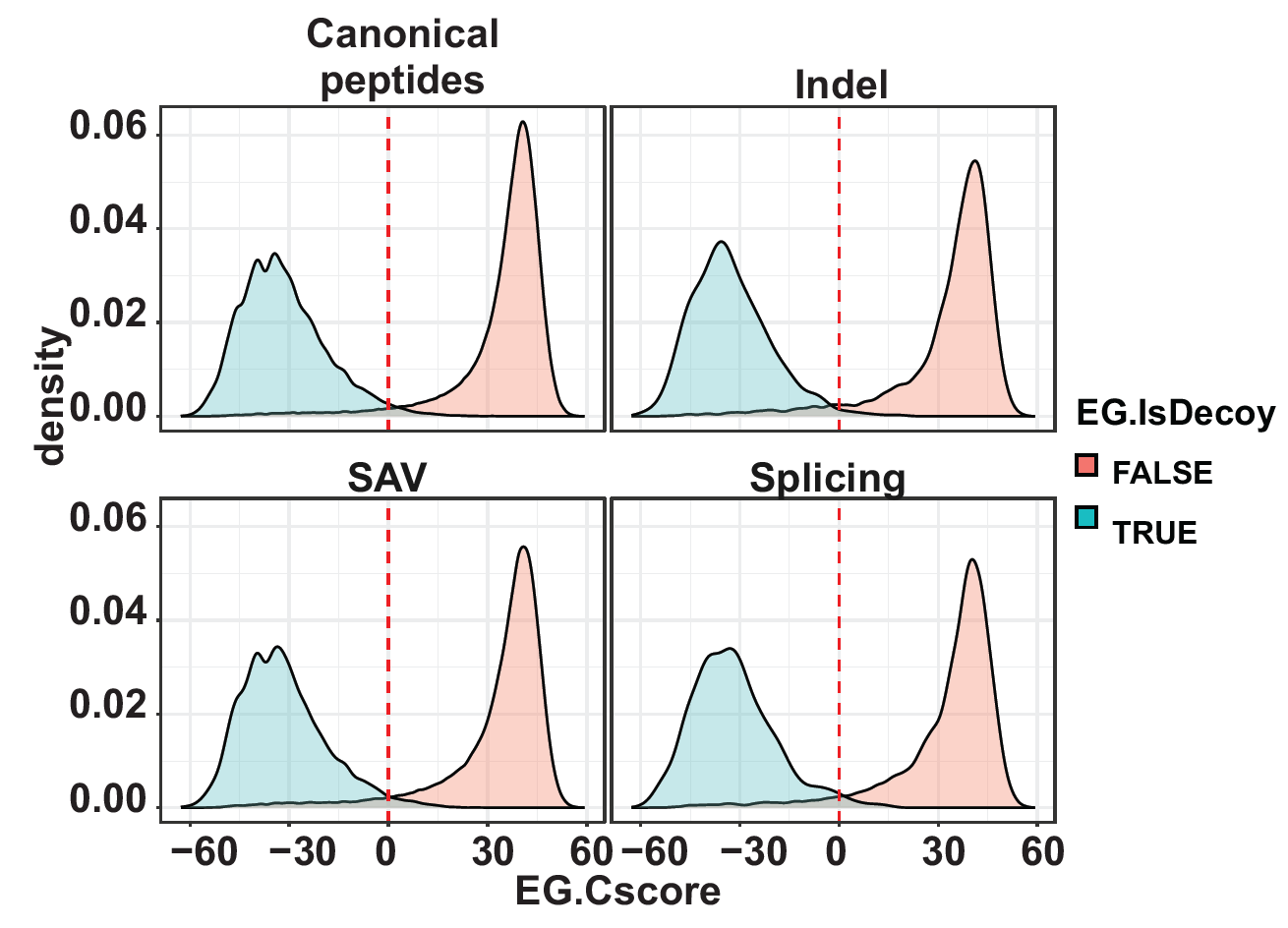
 **Supplementary Fig. 7. Density distribution of EG.Cscore for SAVs, indels, splice variants, and canonical peptides, along with their corresponding decoy peptides.** Distributions of decoy hits are shown in blue, and true hits are shown in red.


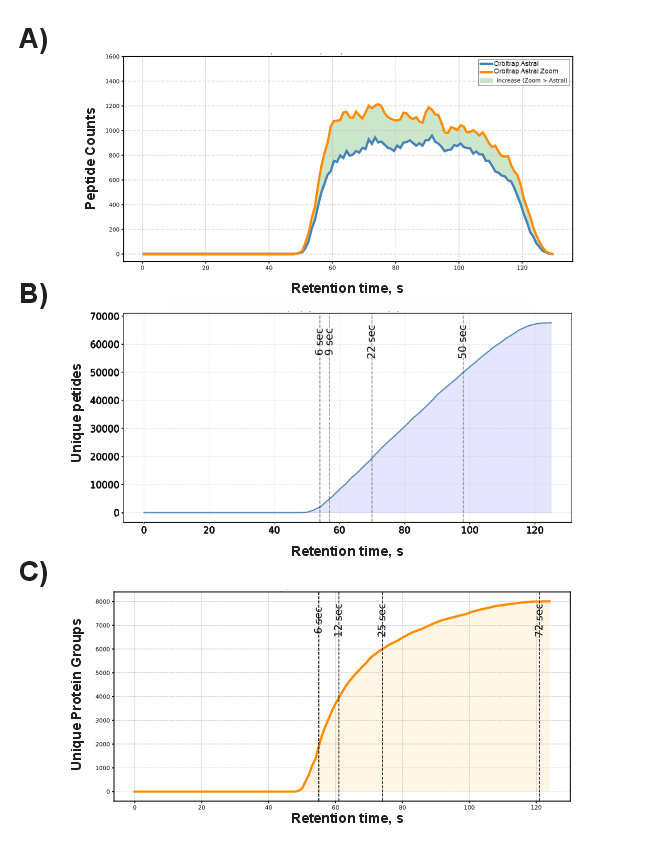


**Supplementary Fig. 8 Proteome depth achieved with high-throughput ultra-fast LC gradients. A)** Number of peptides identified over the course of the 500 SPD LC gradient for the Orbitrap Astral MS (blue) and Orbitrap Astral Zoom MS (orange); time intervals with increased identification rates in the Orbitrap Astral Zoom MS are highlighted in green. **B)** Cumulative number of unique peptides identified across the LC gradient. Dashed lines indicate the time points at which 20,000 and 50,000 unique peptides were detected. **C)** Cumulative number of protein groups identified across the LC gradient. Dashed lines indicate the time points corresponding to the identification of 2,000, 4,000, 6,000, and 8,000 unique protein groups.


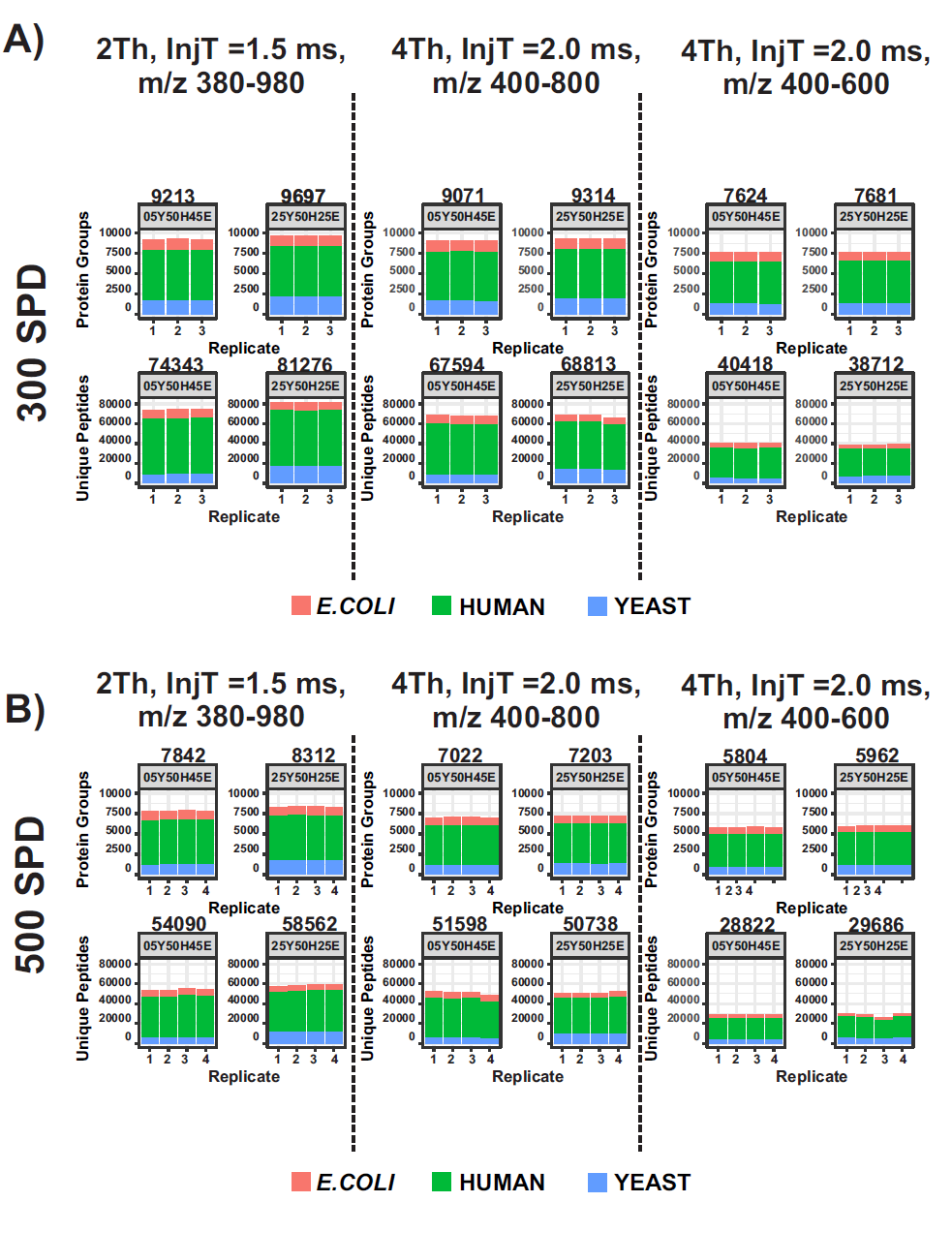


**Supplementary Fig. 9. Protein group and unique peptide identifications across MS acquisition methods and fast LC gradients.** (A) Number of protein groups and unique peptides identified per replicate across three acquisition schemes. Median values are shown above each bar. Bars indicate identifications from human (green), yeast (blue), and E. coli (red). (B) Same as panel A, but for a 500 SPD gradient. Datasets were processed with Spectronaut v19.0.

**
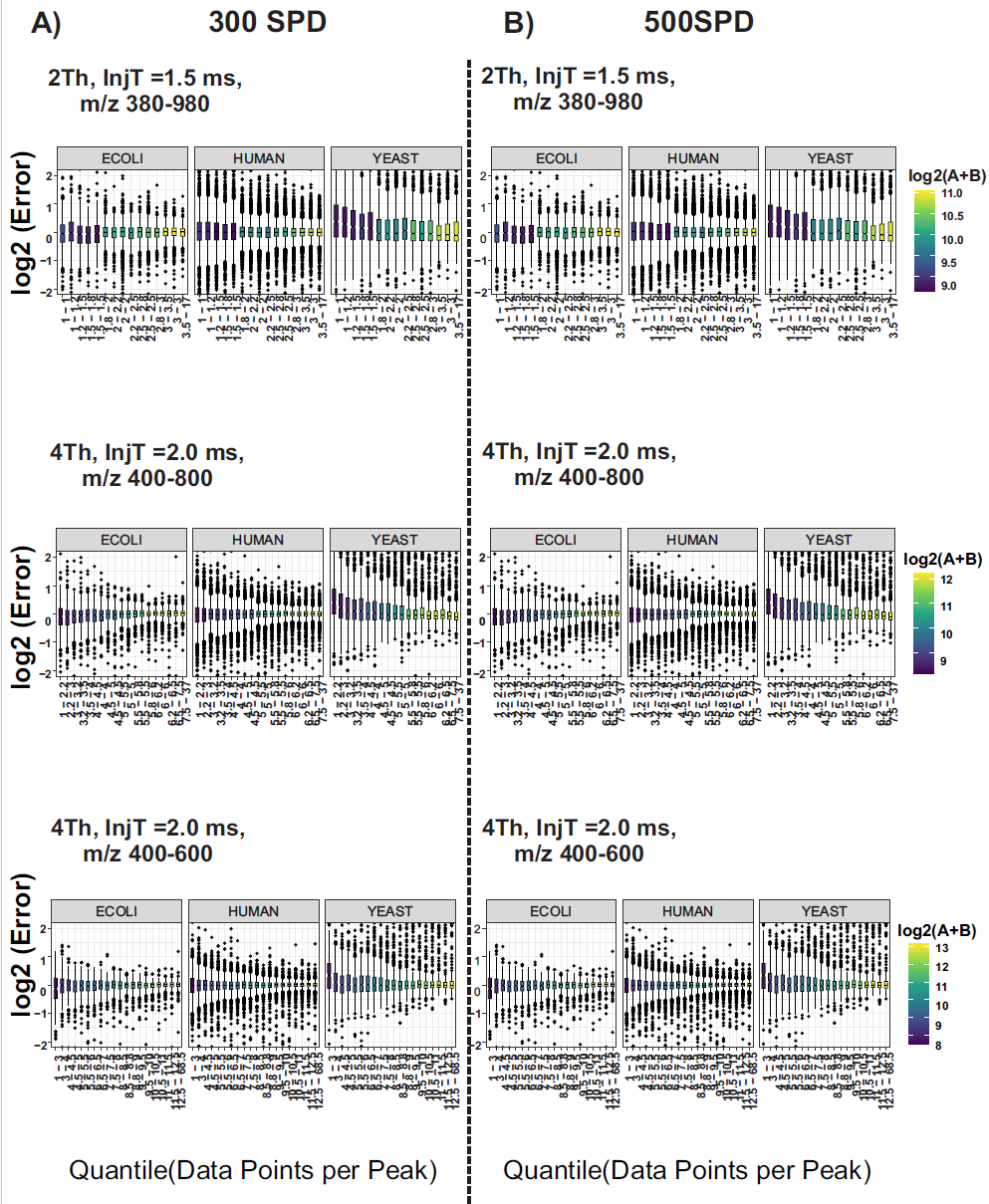
****Supplementary Figure 10. Quantitative accuracy assessed by the log₂(Expected/Empirical).** Log2error as a function of the median number of data points per peak across MS methods, shown for three replicates. (A) Log2 error versus data points per peak for three MS acquisition methods: Reference (2 Th isolation windows, 1.5 ms injection time, m/z 380–980), Method 1 (4 Th isolation windows, 2 ms injection time, m/z 400–800), and Method 2 (4 Th isolation windows, 2 ms injection time, m/z 400–600). (B) Same analysis as in panel A, performed using the ultra-fast 500-SPD LC gradient.

**
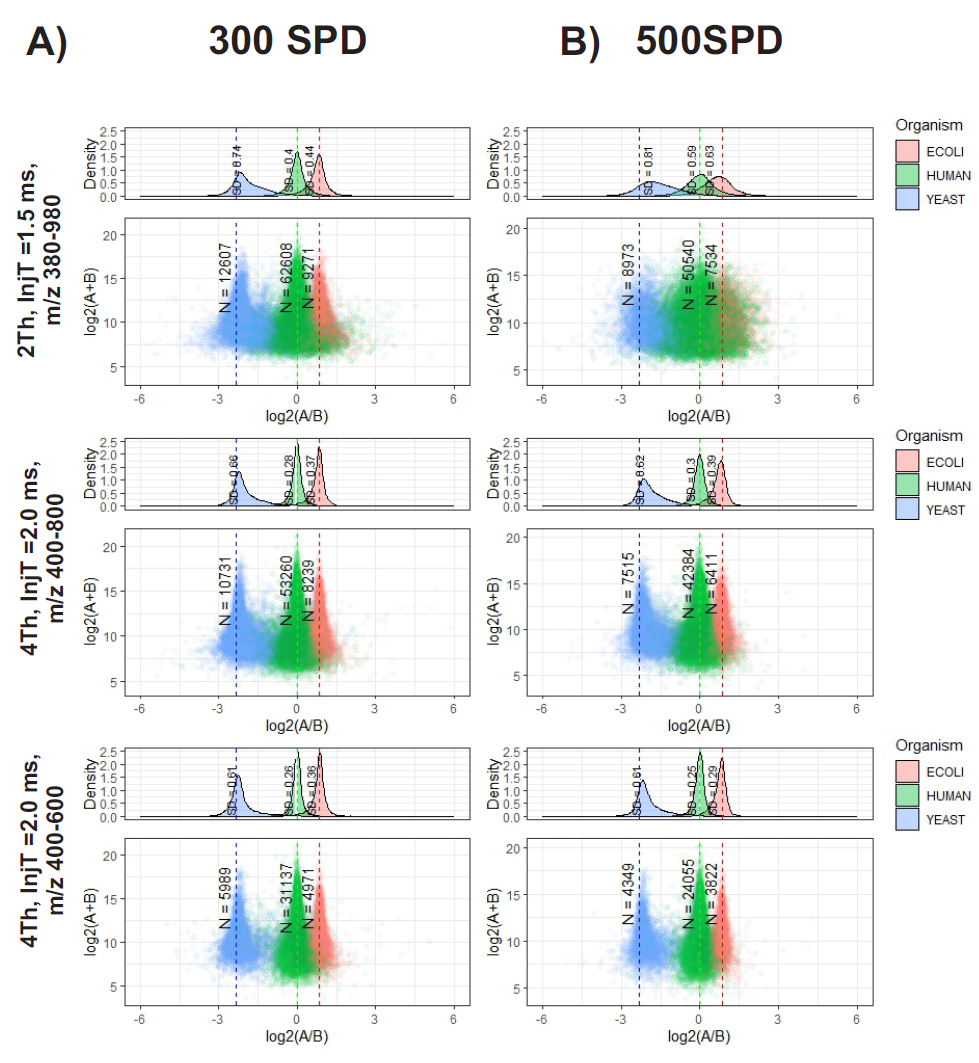
Supplementary Figure 11. Peptide-level assessment of LFQ accuracy and precision on the Orbitrap Astral Zoom MS under different LC gradients and high-speed scanning methods.** (A) Log2-transformed ratios of quantified peptides across scanning methods at a 300 SPD gradient. Density plots are shown at the top, with scatter plots of all runs over log2-transformed peptide intensities at the bottom. Colored dashed lines indicate expected log2-fold-change values for proteins from humans (green), yeast (blue), and E. coli (red). Standard deviations are indicated on the density plots. (B) Same as panel A, but for a 500 SPD gradient. Datasets were processed with Spectronaut v19.0.

**
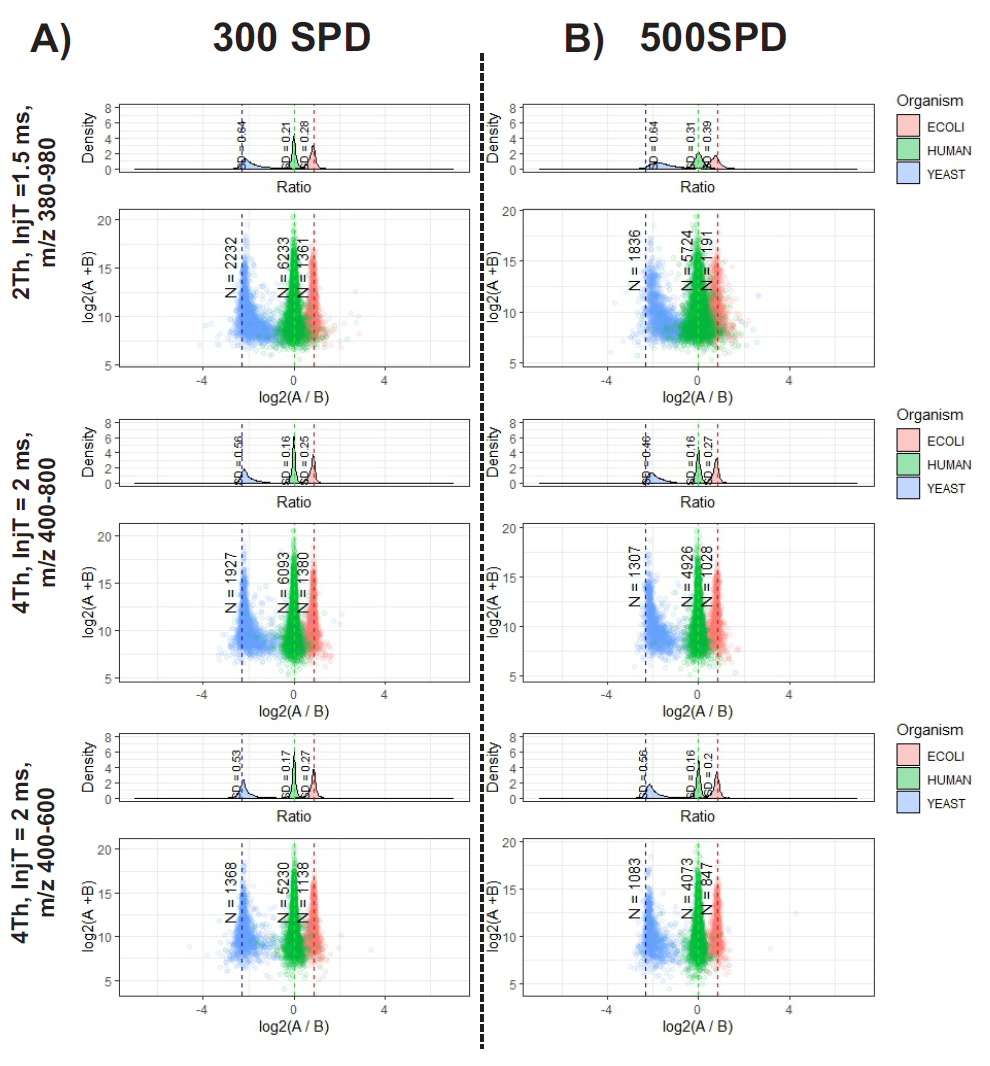
**

**Supplementary Figure 12. Protein-level assessment of LFQ accuracy and precision on the Orbitrap Astral Zoom MS under different LC gradients and high-speed scanning regimes.** (A) Log2-transformed ratios of quantified proteins across scanning methods at a 300 SPD gradient. Density plots are shown at the top, with scatter plots of all runs over log2-transformed protein intensities at the bottom. Colored dashed lines indicate expected log2-fold-change values for proteins from humans (green), yeast (blue), and E. coli (red). Standard deviations are indicated on the density plots. (B) Same as panel A, but for a 500 SPD gradient. Datasets were processed with Spectronaut v19.0.

**
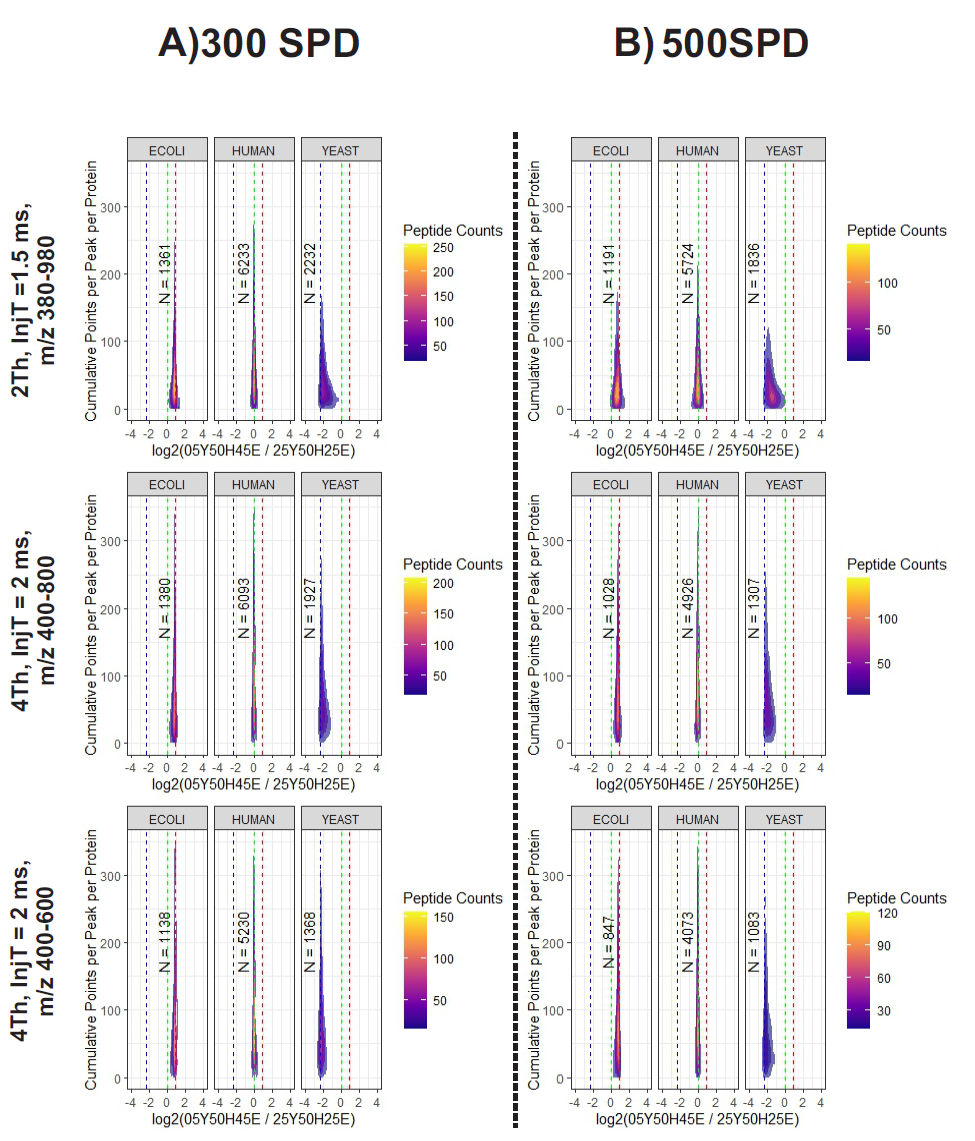
**

**Supplementary Figure 13. Protein-level assessment of LFQ accuracy and precision on the Orbitrap Astral Zoom MS under different LC gradients and high-speed scanning regimes, shown as a function of cumulative points per peak per protein.** (A) Log2-transformed ratios of quantified proteins across scanning methods at a 300 SPD gradient. Scatter plots display protein ratios, with density overlays indicating the number of peptides as a function of cumulative points per peak per protein. Colored dashed lines represent expected log2-fold-change values for proteins from humans (green), yeast (blue), and E. coli (red). Standard deviations are indicated on the density plots. (B) Same as panel A, but for a 500 SPD gradient. Datasets were processed with Spectronaut v19.0.

**
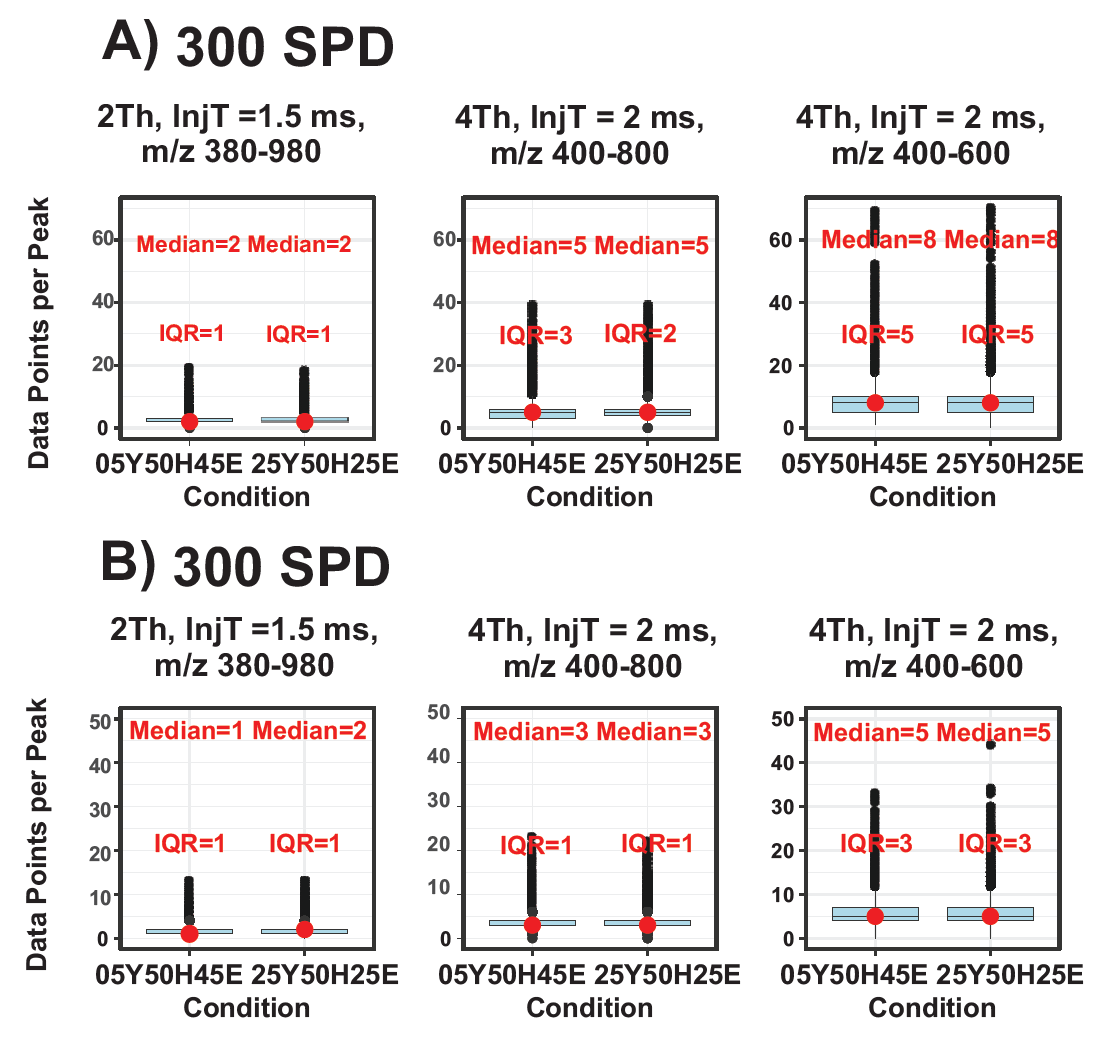
**

**Supplementary Figure 14. Data points per peak (DPPP) across conditions, MS scanning methods, and LC gradients.** (A) Boxplots showing DPPP for different conditions (05Y50H45E and 25Y50H25E) across MS scanning methods using a 300 SPD gradient. (B) Same as panel A, but using a 500 SPD gradient. Datasets were processed with Spectronaut v19.0 n= 3.

**
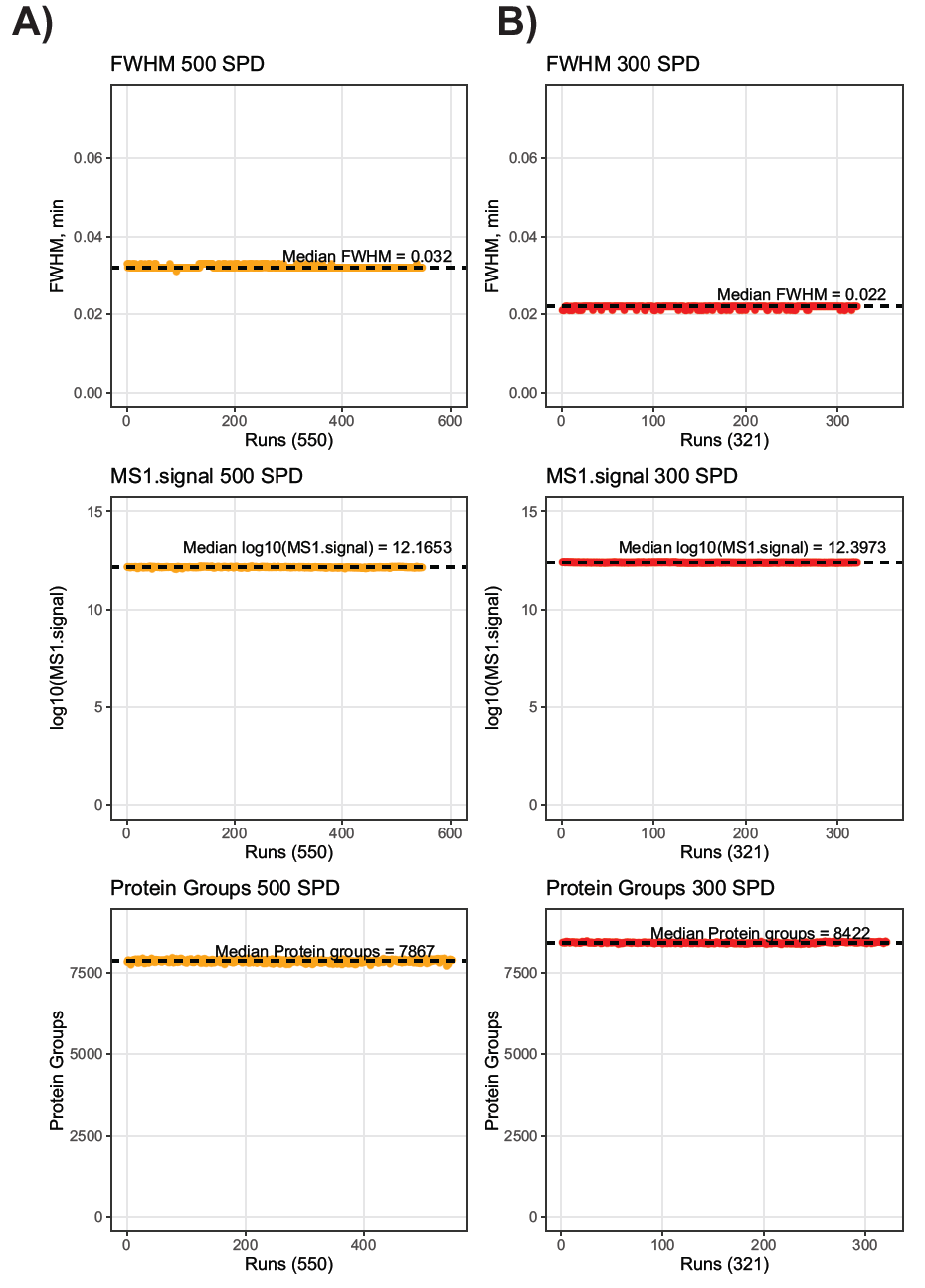
**

**Supplementary Figure 15. Ultra-fast gradient stability tests.** **A)** Stability test for the 500-SPD method showing median FWHM (top panel), log-transformed MS1 signal intensity (middle panel), and total protein group identifications (bottom panel) across >500 consecutive injections. **B)** Same analysis for the 300-SPD method across >300 consecutive injections. For both gradients, 200 ng of HEK tryptic peptides were analyzed per run.


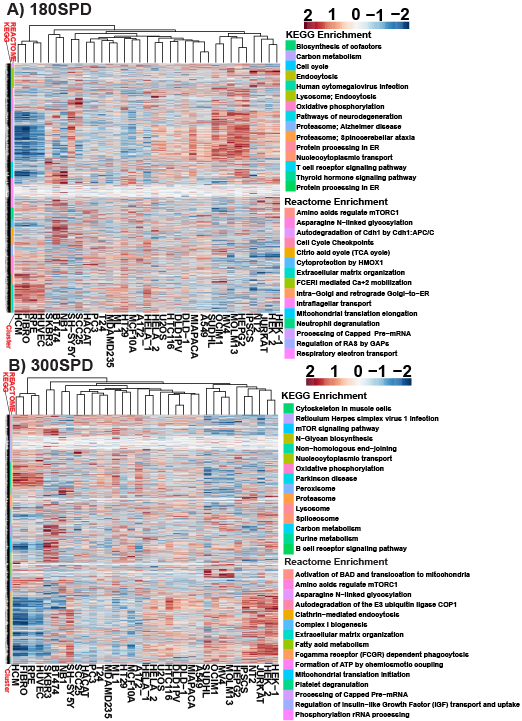


**Supplementary Fig. 16.** **Functional landscape across 32 cell lines. (A)** Hierarchical clustering heatmap of protein expression profiles measured across 32 cell lines at 180 SPD. Bar colors depict the KEGG and Reactome pathway enrichment analyses of the identified protein clusters associated to functional signatures. **(B)** Hierarchical clustering heatmap of protein expression profiles measured across 32 cell lines at 300 SPD. Bar colors depict the KEGG and Reactome pathway enrichment analyses of the identified protein clusters associated to functional signatures.
